# Odorant and Gustatory Receptors in the Tsetse Fly *Glossina morsitans morsitans*

**DOI:** 10.1371/journal.pntd.0002663

**Published:** 2014-04-24

**Authors:** George F. O. Obiero, Paul O. Mireji, Steven R. G. Nyanjom, Alan Christoffels, Hugh M. Robertson, Daniel K. Masiga

**Affiliations:** 1 Molecular Biology and Bioinformatics Unit, International Center of Insect Physiology and Ecology (icipe), Nairobi, Kenya; 2 South African Bioinformatics Institute (SANBI), South African MRC Bioinformatics Unit, University of the Western Cape, Bellville, South Africa; 3 Department of Biochemistry and Molecular Biology, Egerton University, Njoro, Kenya; 4 Department of Biochemistry, Jomo Kenyatta University of Agriculture and Technology, Nairobi, Kenya; 5 Department of Entomology, University of Illinois at Urbana-Champaign, Urbana, Illinois, United States of America; National Institute of Allergy and Infectious Diseases, United States of America

## Abstract

Tsetse flies use olfactory and gustatory responses, through odorant and gustatory receptors (ORs and GRs), to interact with their environment. *Glossina morsitans morsitans* genome ORs and GRs were annotated using homologs of these genes in *Drosophila melanogaster* and an *ab initio* approach based on OR and GR specific motifs in *G. m. morsitans* gene models coupled to gene ontology (GO). Phylogenetic relationships among the ORs or GRs and the homologs were determined using Maximum Likelihood estimates. Relative expression levels among the *G. m. morsitans* ORs or GRs were established using RNA-seq data derived from adult female fly. Overall, 46 and 14 putative *G. m. morsitans* ORs and GRs respectively were recovered. These were reduced by 12 and 59 ORs and GRs respectively compared to *D. melanogaster*. Six of the ORs were homologous to a single *D. melanogaster* OR (DmOr67d) associated with mating deterrence in females. Sweet taste GRs, present in all the other Diptera, were not recovered in *G. m. morsitans*. The GRs associated with detection of CO_2_ were conserved in *G. m. morsitans* relative to *D. melanogaster*. RNA-sequence data analysis revealed expression of GmmOR15 locus represented over 90% of expression profiles for the ORs. The *G. m. morsitans* ORs or GRs were phylogenetically closer to those in *D. melanogaster* than to other insects assessed. We found the chemoreceptor repertoire in *G. m. morsitans* smaller than other Diptera, and we postulate that this may be related to the restricted diet of blood-meal for both sexes of tsetse flies. However, the clade of some specific receptors has been expanded, indicative of their potential importance in chemoreception in the tsetse.

## Author Summary

Tsetse flies navigate their environments using chemosensory receptors, which permit them to locate hosts, mating partners, and resting and larviposition sites. The genome of *G. m. morsitans* was interrogated for coding genes of odorant receptors (ORs) and gustatory receptors (GRs) that express in antennae and maxillary palp, and detect the volatile and soluble chemical signals. The signals are then transmitted to the central nervous system and translated to phenotypes. Majority of these genes in *G. m. morsitans* were spread across different scaffolds, but a few were found to occur in clusters, which suggested possible co-regulation of their expression. The number of ORs and GRs were much reduced in the *G. m. morsitans* genome, including the apparent loss of receptors for sugar when compared to selected Diptera. There was also an apparent numerical expansion of some receptors, presumably to maximize on their restricted blood-meal diet. The annotation of the chemoreceptor package of *G. m. morsitans* provides a resource for investigating key activities of tsetse flies that could be exploited for their control.

## Introduction

Trypanosomiasis management has been a longstanding development preoccupation in sub-Saharan Africa, with tsetse fly control constituting the cornerstone in this effort [1]. Since all tsetse species are able to transmit trypanosomes, the critical determinant of transmission is their obligate blood feeding. Tsetse flies select their hosts through visual and olfactory signals, a process that is mediated by olfactory and gustatory receptors. Tsetse flies navigate their environment by detecting and responding to volatiles and non-volatile cues (odors and tastants). Artificial bait technologies, based on tsetse olfactory responses to natural cues and blends of synthetic versions that mimic those of their natural hosts in the field, have successfully been applied in tsetse control because of their relatively high specificity, low cost, community acceptability, and ability to slow down tsetse re-invasion from adjacent areas [2], [3]. These technologies are environment friendly [4], and applicable for riverine and savanna species of tsetse flies [5], [6]. The attractants include various phenolic derivatives [7]–[9], carbon dioxide, acetone, 1-octen-3-ol, and vertebrate host breath, skin and urine extracts [10]–[12]. Interestingly, 1-octen-3-ol is a constituent of the chemical profile from *Lantana camara*, an invasive plant to which tsetse flies are attracted [13]. The response to olfactory cues has also been exploited in design of tsetse repellents [14], [15]. The repellents include guaiacol (methylphenols), δ-octalactone and methylketones [16]–[18] and 2-methoxy-4-methylphenol [14]. Natural differential responses among tsetse species and even between sexes and allopatric populations of the same species have been observed [18]–[22], which have stimulated research and design to enhance the efficiencies of the existing attractant-based bait technologies, to develop new ones based on repellent blends (‘push’ tactics) from refractory animals, and to integrate these into ‘push-pull’ strategies. Different *Glossina* species exhibit different olfactory uniqueness' and this may partly account for the observed graduation of preferences for particular hosts. For instance, riverine tsetse species (such as *G. fuscipes fuscipes*, *G. palpalis* and *G. tachinoides*) prefer feeding on reptilian hosts compared to their savanna relatives (*G. morsitans morsitans*, *G. pallidipes*) that feed largely on ungulates and other large mammals [6]. Larvipostion pheromones (n-pentadecane and n-dodecane) from exudates of mature larvae are also known to attract and induce gravid *G. m. morsitans* and *G. m. centralis* females to aggregate and deposit larvae [23]. Research on response to tastants in tsetse flies are limited, but point to their potential application in tsetse control [10], [24]. In all, responses to odors and tastants in tsetse have established utility in tsetse control that can be augmented with better understanding of the molecular factors that underpin these responses.

Molecular factors mediating the olfactory and gustatory responses in the tsetse flies are poorly understood. However, research on other insects indicates that the odors and tastants in the environment are generally detected in peripheral sensory neurons by distinct odorant and gustatory receptors (ORs and GRs) [25]–[28]. These receptors are divergent members of a superfamily characterized by seven transmembrane domains, and share low sequence conservation among them except at the C-terminus region that coincides with the seventh trans-membrane domain [29]. The ORs and GRs are thought to have evolved as parallel chemoreceptors across diverse organisms [26]. Each OR is expressed in olfactory receptor neurons (ORNs) within maxillary palpi and antennae [25], [30]–[32]. The ORs generally have multiple introns and are very divergent with poor structural conservation within and across insect orders and species [33], [34], which potentially reflect diverse olfaction related preferences within the orders and species. However, a canonical co-receptor commonly referred to as Orco remains highly conserved across insect orders [35]–[38], a phenomenon that may be associated with its role in proper tuning of odor specificity and activation necessary for appropriate signal transduction in the neurons [39]. The GRs are generally expressed in gustatory receptor neurons (GRNs) within gustatory organs [40] in response to soluble taste and contact pheromones [41], [42]. However, some GRs are expressed in antennal dendrites and respond to carbon dioxide, potentially implicating them in olfaction [40], [43]. The GRs are more conserved in sequence and structure than the ORs [44], [45] probably due to comparatively smaller search space among cues associated with GRs than ORs. The diversity among the ORs and GRs in tsetse can potentially shed light on the natural differential responses observed among them [12], [17], [18], [20]–[29], with potential application in tsetse control. To improve or develop new approaches of vector management, an understanding of the molecular attributes of GRs and ORs and their potential roles in tsetse ecology is essential.

This study was initiated to (1) comparatively annotate and catalogue ORs and GRs in *G. m. morsitans* (GMOY1.1), (2) establish evolutionary distance between *G. m. morsitans* ORs or GRs and those in especially *D. melanogaster*, and (3) examine relative expression of the ORs and GRs in the *G. m. morsitans*. The assembly has been estimated to be over 99% complete based on the software Core Eukaryotic Genes Mapping Approach (CEGMA) [46] and manually sequenced BACs data. The assembly is currently undergoing genome-wide manual curation and annotation by the International Glossina Genome Initiative (IGGI) consortium.

## Methods

### Retrieval and annotation of *G. m. morsitans* OR and GR gene models

Coding sequences (CDS) of ORs and GRs in *Drosophila melanogaster* were obtained from FlyBase5.13 [47] and used to isolate their respective homologs in the *G. m. morsitans* genome (GMOY1.1) at VectorBase [48] using tBLASTx algorithm [49]. Scaffolds encoding the homologs were searched for and retrieved at a cut-off e-value <1.0e-05. Whole transcriptome illumina 84 million RNA sequence reads generated from female *G. m. morsitans* [50] were mapped onto the scaffolds using default settings in CLC Genomics workbench suite Version 4.8 (CLC Bio, Aarhus, Denmark). Gene loci of putative *Glossina* homologs were curated in the scaffold sequences flanking the tBLASTx hits, and intron/exons modeled using the RNA-seq mappings. The predicted gene models were viewed and edited using Artemis v13.2.12 [51] where, intron/exon boundaries were edited using motifs GT for 5′ donor site, and AG for 3′ acceptor site. The start codon (ATG) for each gene model was fixed at the 5′ end and the reading frame terminated at the first of any of the stop codons (TAA, TGA, or TAG). Sequences shorter than average size of known insect ORs (370 aa) were marked as incomplete if they lacked start or stop codons. Sequences with poorly conserved functional domains were considered as pseudogenes.

The homologs were validated through sequence-based searches for presence of ORs or GRs specific 7tm-6-olf-recpt or 7tm-7-olf-recpt [29], [52] domains respectively. The homologs were probed for the domains using DELTA BLAST algorithm [53] against the conserved domains databases (CDD) [54], and presence of alpha helix trans-membrane domains validated using TMHMM server v2.0 [55]. Additionally, all the putative ORs or GRs were validated, using BLAST2GO analyses [56] against the non-redundant Swiss-Prot database [57]. The curated gene models were assigned annotation identifiers by comparing them with automated transcript feature models obtained from the Glossina community annotation portal at VectorBase [48] and edited using Artemis genome viewer tool [51]. The models without automated prediction matches and identifiers were manually built using the Artemis gene build tool window [51] and given unique temporary annotation identifiers. In this respect, features for gene, exons, mRNA, and CDS were created for such gene models. The Glossina gene models were assigned putative gene names where GmmOR* and GmmGR* were adopted for *G. m. morsitans* odorant receptors and gustatory receptors respectively (the asterisk (*) being an identifier number). The annotated gene model features were submitted to the VectorBase community annotation portal for *G. m. morsitans* [48] for integration into genome database; nevertheless, a list of annotated amino acid coding sequences is presented in supplementary Dataset S1, and a list of associated gene identities in Table S2. The *G. m. morsitans* receptor repertoires were evaluated against those documented for *D. melanogaster*, *Anopheles gambiae*, *Aedes aegypti*, *Apis mellifera*, *Nasonia vitripennis*, *Camponotus floridanus*, *Harpegnathos saltator* and *Tribolium casteneum* (references in Table 1).

**Table 1:** Annotated ORs and GRs in *G. m. morsitans* and other selected insect species.

Insect	ORs	GRs	Reference
*D. melanogaster*	60 (2)*	60 (13)*	[25]–[27], [29], [42]
***G. m. morsitans***	**46 (3)**	**14**	**This study.**
*An. gambiae*	79	76	[52], [76]
*Ae. aegypti*	100(31)	79	[74], [77]
*Apis mellifera*	163 (11)	10 (3)	[41]
*Nasonia vitripennis*	225 (76)	47 (11)	[80]
*Camponotus floridanus*	352 (55)	46 (17)	[75]
*Harpegnathos saltator*	347 (30)	17 (4)	[75]
*Tribolium casteneum*	265 (76)	220 (25)	[78], [79]

Figures in parentheses are numbers incomplete genes and or pseudogenes of the receptors.

*- in parentheses are alternatively spliced forms.

### Phylogenetic analyses of ORs and GRs in *G. m. morsitans* and selected Diptera

MUltiple Sequence Comparison by Log-Expectation (MUSCLE) tool [58] was used to align GmmORs and GmmGRs with homologs in *D. melanogaster*, and the alignments edited using Jalview web-server [59]. The secondary structures in the alignments were predicted using JPred program [60]. Phylogenetic cluster inference was done using Maximum Likelihood approach with best fitting Wheelan and Goldman+Freq (WAG+F) model [61], which was chosen as the best ranked from a panel of all amino acid model tests run in MEGA5 [62]. The initial tree was automatically generated and bootstrapped with 500 iterations. The evolutionary rate difference among sites was modeled using a discrete Gamma distribution (5 categories (+G, parameter = 4.2651)). The rate variation model allowed for some sites to be evolutionarily invariable ([+I], 0.8705% sites). All positions with less than 95% site coverage were eliminated and branch nodes determination set at very strong. Evolutionary analyses were conducted using the MEGA5 suite [62].

### Comparative analyses of expression profiles of *G. m. morsitans* ORs and GRs

The expression profiles of *G. m. morsitans* ORs and GRs gene loci were determined using whole transcriptome 84 million illumina RNA-sequence reads [50]. The RNA-seq reads were mapped onto the *G. m. morsitans* ORs or GRs nucleotide coding sequences (CDS) in CLC Genomics Workbench (CLC Bio, Aarhus, Denmark) via RNA-seq analysis pipeline with default settings. The expression profiles were presented as reads per kilobase of exon model per million mapped reads (RPKM) for each receptor sequence [63].

## Results

Most of the gene loci of *G. m. morsitans* ORs and GRs were scattered amongst the scaffolds. Fifty percent of *G. m. morsitans* OR genes were encoded as single-copies on their respective scaffolds. The remainder were encoded in pairs or triplets per scaffold. Five *G. m. morsitans* OR loci (GmmOR6/7/8, GmmOR18/19, GmmOR22/25, GmmOR27/28 and GmmOR41/42) were located in tandem on their respective scaffolds. Similarly, five *G. m. morsitans* GR genes clustered on a single scaffold. The rest were encoded as single-copies on their respective scaffolds. All *G. m. morsitans* GR loci were annotated as complete genes.

### Gene models for *G. m. morsitans* OR and GR and their annotation

Numbers of OR and GR gene loci recovered in *G. m. morsitans*, relative to those published in other insects are summarized in Table 1. Similar to most insects, the *G. m. morsitans* has more ORs loci than GRs loci, with the exception of *D. melanogaster* where the numbers are equal. However, the *G. m. morsitans* ORs are fewer than those documented in all the insects evaluated, including *D. melanogaster*. A similar trend was exhibited in *G. m. morsitans* GRs, except in relation to *A. mellifera*. Annotation of *G. m. morsitans* ORs and GRs are summarized in Table 2. The lengths of *G. m. morsitans* ORs varied between 260 and 541 amino acids, while those of *G. m. morsitans* GRs ranged from 309 to 514 amino acids. The number of exons ranged between two and eight or 12 in GRs and ORs respectively. The predicted genome structures are given in Figure S1. The frequency of detectable trans-membrane domains was also variable, with proteins having six trans–membrane domains representing about one half of all genes. The *G. m. morsitans* ORs (57%, 26 out of 46) were homologous to nine *D. melanogaster* ORs. Similarly, most of the *G. m morsitans* GRs (57%, 8 out of 14) were homologous to three *D. melanogaster* GRs genes. The remainder of the *G. m. morsitans* GRs had one-to-one homology with a single *D. melanogaster* specific homolog. Reciprocal blasts onto non-redundant protein databases for both *G. m. morsitans* ORs and GRs are summarized in Supplementary material – Table S1). GmmGR3 and GmmGR4 were also homologous to *An. gambiae* orthologs, while GmmGR5, GmmGR8 and GmmGR13 had homologs to genes in other *Drosophila* species. The *G. m. morsitans* ORs pseudogenes were scanty, representing 7% of the ORs genes recovered. Only GmmOR5 had alternative splice variants. The 7tm-6-olfct-rcpt domain was detected in all *G. m. morsitans* ORs, and the 7tm-7-chem-rcpt domain was detected in five ORs (GmmOR17, GmmOR21, GmmOR24, GmmOR38 and GmmOR39). The 7tm-7-chem-rcpt domain was also detected in all the *G. m. morsitans* GRs.

**Table 2:** Annotations of odorant and gustatory receptor genes in *G m. morsitans* and their homologs in *D. melanogaster*.

*G. m. morsitans* Genes	Length (AA)	Exons	TMMs	Gene ID	*Dmel* orthologs/Accession Number
GmmOR1	521	8	6	GMOY005610	DmOr83b/CG10609
GmmOR2	394	3	7	GMOY005796	DmOr2a/CG3206
GmmOR3	387	3	3	GMOY004772	DmOr19a/CG18859
GmmOR4	384	2	7	TMP_Or4	DmOr59a/CG9820
GmmOR5*	442	4	5	GMOY012018	DmOr33b/CG16961
GmmOR6	387	4	5	GMOY009475	DmOr42b/CG12754
GmmOR7	406	3	6	TMP_Or7	DmOr42b/CG12754
GmmOR8	389	4	6	TMP_Or8	DmOr42b/CG12754
GmmOR9	409	3	6	TMP_Or9	DmOr42b/CG12754
GmmOR10	444	3	6	TMP_Or10	DmOr46a/CG33478
GmmOR11	341	3	6	GMOY010761	DmOr46a/CG33478
GmmOR12	340	3	3	GMOY009271	DmOr94b/CG17241
GmmOR13	391	6	6	GMOY003312	DmOr82a/CG31519
GmmOR14	341	3	6	GMOY001365	DmOr45a/CG1978
GmmOR15	446	4	7	TMP_Or15	DmOr45a/CG1978
GmmOR16	387	4	6	TMP_Or16	DmOr45a/CG1978
GmmOR17	541	12	8	GMOY005386	DmOr69a/CG33264
GmmOR18	420	8	6	TMP_Or18	DmOr63a/CG9969
GmmOR19	385	8	7	GMOY012322	DmOr63a/CG9969
GmmOR20#	269	7	6	TMP_Or20	DmOr85b/CG11735
GmmOR21	465	5	2	GMOY011399	DmOr83a/CG10612
GmmOR22#	296	4	5	TMP_Or22	DmOr49a/CG13158
GmmOR23	331	4	5	TMP_Or23	DmOr85b/CG11735
GmmOR24	388	3	6	GMOY010839	DmOr85c/CG17911
GmmOR25	385	3	6	GMOY012357	DmOr56a/CG12501
GmmOR26	418	4	5	TMP_Or26	DmOr85b/CG11735
GmmOR27	415	3	6	GMOY008038	DmOr67c/CG14156
GmmOR28#	260	2	7	TMP_Or28	DmOr92a/CG17916
GmmOR29	438	3	4	TMP_Or29	DmOr67a/CG12526
GmmOR30	361	3	6	TMP_Or30	DmOr67a/CG12526
GmmOR31	435	7	5	TMP_Or31	DmOr24a/CG11767
GmmOR32	450	5	7	GMOY005084	DmOr13a/CG12697
GmmOR33	353	6	5	GMOY005479	DmOr49b/CG17584
GmmOR34	360	7	4	GMOY011902	DmOr30a/CG13106
GmmOR35	392	5	6	TMP_Or35	DmOr43a/CG1854
GmmOR36	343	7	6	TMP_Or36	DmOr43a/CG1854
GmmOR37	430	4	4	TMP_Or37	DmOr74a/CG13726
GmmOR38	371	5	6	TMP_Or38	DmOr47b/CG13206
GmmOR39	403	3	6	GMOY004392	DmOr88a/CG14360
GmmOR40	284	5	6	GMOY012356	DmOr56a/CG12501
GmmOR41	386	4	6	GMOY006480	DmOr67d/CG14157
GmmOR42	386	4	5	GMOY006479	DmOr67d/CG14157
GmmOR43	389	4	5	TMP_Or43	DmOr67d/CG14157
GmmOR44	390	4	6	GMOY006265	DmOr67d/CG14157
GmmOR45	385	4	7	GMOY007896	DmOr67d/CG14157
GmmOR46	348	4	3	GMOY003305	DmOr67d/CG14157
GmmGR1	425	3	6	GMOY007472	DmGr21a/CG13948
GmmGR2	514	7	6	GMOY011510	DmGr22b/CG31931
GmmGR3	425	6	6	TMP_Gr5	DmGr21a/CG13948
GmmGR4	496	8	6	GMOY008001	DmGr63a/CG14979
GmmGR5	467	5	7	GMOY004207	DmGr66a/CG7189
GmmGR6	443	4	8	GMOY011615	DmGr28b/CG13788
GmmGR7	402	3	7	GMOY006209	DmGr28b/CG13788
GmmGR8	407	2	6	TMP_Gr4	DmGr22e/CG31936
GmmGR9	348	5	4	GMOY011903	DmGr2a/CG18531
GmmGR10	458	4	7	GMOY003231	DmGr33a/CG17213
GmmGR11	450	3	6	TMP_Gr3	DmGr22b/CG31931
GmmGR12	375	2	8	TMP_Gr2	DmGr32a/CG14916
GmmGR13	457	2	6	TMP_Gr1	DmGr22b/CG31931
GmmGR14	309	3	6	TMP_Gr6	DmGr22b/CG31931

GmmOR – *Glossina morsitans morsitans* ordorant receptor; GmmGR- *G. m. morsitans* gustatory receptor; TMM- Trans-membrane helices; GMOY – *Glossina morsitans* Yale strain; TMP_Or – Provisional odorant receptor ID; TMP_Gr – Provisional gustatory receptor ID; DmOr- *Drosophila melanogaster* odorant receptor; DmGR- *D. melanogaster* gustatory receptor;

*- longest alternative splice variant in locus OR5;

# - pseudogene.

### Phylogenetic analysis of *G. m. morsitans* ORs and GRs with other insects

**Figure 1:**
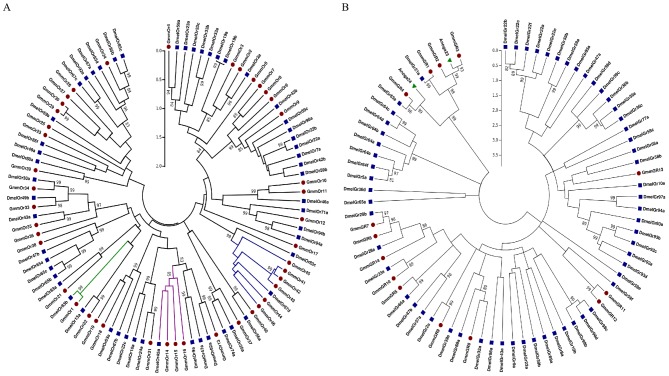
Phylogenetic analyses of ORs or GRs in *G. m. morsitans* and selected Diptera. (**A**) Maximum likelihood (ML) tree for GmmORs and DmelOrs; branches annotated blue is an expanded clade orthologous to DmelOr67d; purple branches is the clade orthologous to DmelOr45a; and green branches indicate the orco cluster. (**B**) Maximum likelihood tree for GmmGRs and DmelGRs. In both trees, blue labels are *D. melanogaster* receptors and red labels *G. m. morsitans* receptors (green labels are *An. gambiae* CO2 receptors). Phylogenetic cluster inferences were deduced using Maximum Likelihood approach with best fitting Wheelan And Goldman+Freq (WAG+F) model [59]. Evolutionary analyses were conducted using MEGA5 suite [60].

Phylogenetic relationships between *G. m. morsitans* ORs and GRs and their counterparts in *D. melanogaster* are summarized in Figure 1. Most of the *G. m. morsitans* ORs and GRs clustered with their respective ORs and GRs orthologs with a bootstrap support of over 80%. The *G. m. morsitans* OR14, OR15 and OR16 were homologous to a drosophila larvae receptor, Or45a. The *G. m. morsitans* co-receptor (Orco) (GmmOR1) had 100% bootstrap support homology to *D. melanogaster* homolog, Or63b, and was a single copy in the genome, similar to other insects investigated (data not shown). There was an expanded cluster of ORs in *G. m. morsitans* (GmmOR41-46), relative to a single *D. melanogaster* homolog, Or67d (Figure 1A), which also had multiple copies in *An. gambiae*, *Cu. quinquefasciatus*, *Ae. aegypti*, *Tribolium casteneum* (Data not shown). The *G. m. morsitans* and *D. melanogaster* GRs clustered into four groups (Figure 1B). Four *G. m. morsitans* GRs (GmmGR1-4) clustered with homologs of CO_2_ receptors, Gr21a and Gr63a in *D. melanogaster*; GmmGR6-7 and GmmGR14, though distantly, clustered with an unusual splice variant DmelGr28a/28b; GmmGR5, 8–12 were homologous to bitter taste-related sensors in *D. melanogaster*; and GmmGR13 clustered distantly to DmelGr58a/58b homologs, whose functions are unknown.

### Relative expression profiles of *G. m. morsitans* ORs and GRs

**Figure 2:**
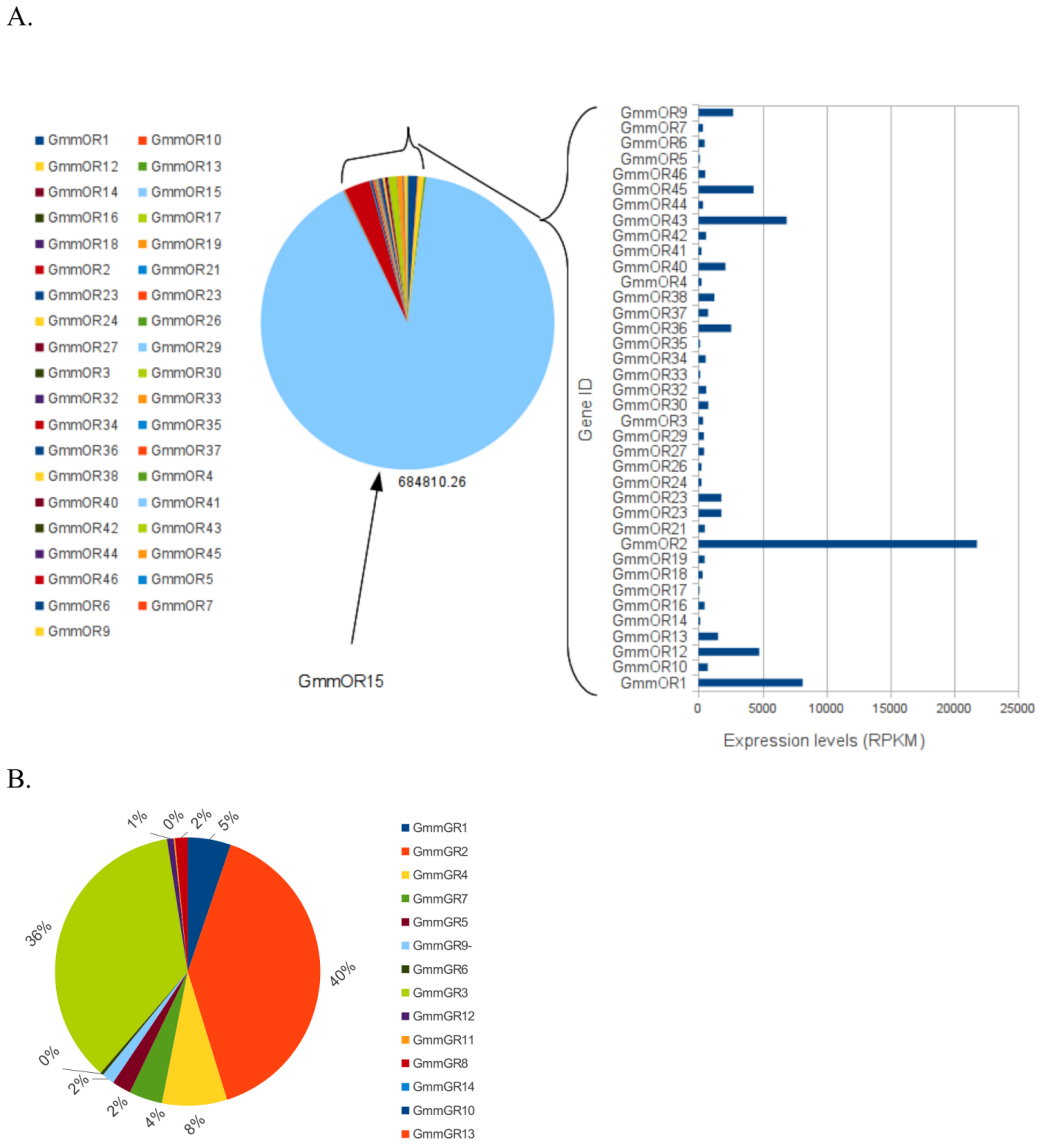
Glossina chemoreceptor expression abundances by RNA-seq data in RPKM. (**A**) Expression abundances of *GmmORs*. There was no sufficient data to support profiles for GmmOR8, 11, 25, 31, and 39. GmmOR15 had abundant transcriptome data of 90.746% relative to sequence reads that mapped onto *GmmORs*. (**B**) Expression abundances of *GmmGRs*. Expression profiles for GmmGR6, GmmGR11 and GmmGR13 were not detected; GmmGR2 and GmmGR3 accounted for 40% and 36% respectively of the total transcripts considered for *GRs*.

Relative expression profiles of the *G. m. morsitans* ORs and GRs gene loci are summarized in Figure 2. Among the *G. m. morsitans* ORs, expression of *GmmOR15* was surprisingly most predominant, accounting for more than 90% of the total RNA-sequence data supporting expression of the ORs. *GmmOR15* is homologous to Or45a gene in *D. melanogaster*. About 5% of RNA-sequence data provided supporting evidence for expression of *GmmOR2*, *GmmOR1* (Orco homolog), *GmmOR43* and GmmOR*9*. Expressions of *GmmOR8*, *GmmOR11*, *GmmOR25*, *GmmOR31*, and *GmmOR39* were not detected in the available RNA-sequence dataset (Figure 2A). Amongst the GRs, *GmmGR1-4* had the best RNA-sequence data expression support (Figure 2B).

## Discussion

Specific groups of the *G. m. morsitans* ORs and GRs were clustered within selected scaffolds. Similar clusters of genes performing common and related functions have been observed among chemosensory genes in *D. melanogaster* [41], [42], [44], and more recently among twelve *G. m. morsitans* major milk proteins associated with lactation [50]. Since genes within clusters are generally co-regulated and can lead to joint gene expression [29], [34], [64], the individual clusters of ORs and GRs might be under common regulatory mechanisms and in response to common or related stimuli. The ORs and GRs in *G. m. morsitans* were fewer than those documented in most insects evaluated (Table 1) [65], [66]. Additionally, specific ORs and GRs in *D. melanogaster* (nine and three ORs and GRs respectively) appear to have been expanded in *G. m. morsitans*, representing more than half of the chemoreceptors.

The factors underlying the apparent reductions and expansions of these receptors in the tsetse are unknown. However, it can be postulated that the obligate blood feeding of the tsetse fly (restricted to vertebrate hosts) relative to *D. melanogaster* (with expansive fruit species hosts) might have necessitated evolutionary selection for specific chemoreceptor loci relevant to discriminate among limited host choices. We know also that environmental factors can determine host choice, as tsetse have been shown to have an acquired preference to specific hosts encountered early in life [67]. Notably, other blood-feeders, such as mosquitoes also seek a variety of plant sources for sugar as energy source, while tsetse flies derive their energy from the amino acids proline and alanine [68]. The *G. m. morsitans* OR15 (GmmOR15) accounted for more than 90% of the OR expression data. This OR is homologous to DmelOr45a, whose product has been, associated with an escape response in *D. melanogaster* larvae [69]. The function of this OR in tsetse was not determined; nonetheless it is notable that the source of RNA sequence data was a reproductively active adult female. Hence, it is possible that the GmmOR15 is in some way associated with larval activity.

Similarly, the GmmGR1-4 cluster was most prominent among the GRs homologous to CO_2_ receptors in *D. melanogaster*. These GRs may be associated with host seeking and may have a duplicate role in olfaction. These receptors may putatively be associated with attractive responses elicited by the savanna tsetse species, including *G. m. morsitans* [10]. From the foregoing, it is evident that tsetse seems to prioritize and invest on a select few chemoreceptor genes towards their adaptive behaviors. Indeed, a heavy investment in specific genes is not uncommon in insects [70]–[73]. The *G. m. morsitans* OR1 (homologous to *Orco*) was the most conserved amongst the *G. m. morsitans* ORs, not surprising since such conservation has been observed in other insects [74] probably due to its critical role in modulating responses of the other receptors.

In conclusion, when examined against other blood feeders, which also take sugar sources from plants (e.g. *An. gambiae* and *Ae. aegypti*), the *G. m. morsitans* has a reduced repertoire of ORs and GRs genes. There is a complete loss of receptors for sugar, and a heavy investment in some chemoreceptors, such as those associated with detection of CO_2_. These observations offer opportunities to develop control tools exploiting these unique adaptations.

## Supporting Information

Dataset S1 **Annotated amino acid sequences of** ***Glossina morsitans morsitans*** **ORs and GRs.** Each sequence name is followed by annotation identity (previous temporary identity), scaffold number and the coordinates within the scaffold.
(PDF)

Figure S1 ***Glossina m. morsitans*** **ORs and GRs genome structure.** Most of the gene loci were encoded as singlets on their respective scaffolds. Some loci were encoded in tandem in their respective scaffolds suggestive of possible joint expression regulation. All genes had multiple exons ranging from two to eight or 12 in GRs or ORs respectively.
(PDF)

Table S1 **Reciprocal blast of** ***G. m. morsitans*** **ORs and GRs to non-redundant protein database at NCBI.** The names of annotated gene loci, scaffold identity, gene location within the scaffold, in bracket (*) refers to the coding strand where (−), reverse and (+), forward strands. The reciprocal blast gives the distribution of homology to other insects including *D. melanogaster*.
(PDF)

Table S2 **List of** ***Glossina m. morsitans*** **gene names and their associated identities.** The vectorbase identities (GMOY….) has since replaced the Glossina community annotation identities (TMP0….) that were computationally derived. Both annotation identities and phylomedb identities can be used as seed queries to retrieve their related Glossinia phylomedb 182 trees (www.phylomedb.org/?q=user/28).
(PDF)

## References

[1] Elliott M, Janes NF, Potter C (1978) The Future of Pyrethroids in Insect Control. Annual Review of Entomology 23: 443–469.

[2] Vale GA, Lovemore DF, Flint SCG (1988) Odour-baited targets to control tsetse flies, *Glossina spp.* (Diptera: Glossinidae) in Zimbabwe. Bull Ent Res 78: 31–49.

[3] Mangwiro TNC, Torr SJ, Cox JR, Holloway M (1999) The efficacy of various pyrethroid insecticides for use on odour-baited targets to control tsetse. Med Vet Ent 13: 315–323. 10.1046/j.1365-2915.1999.00165.x 10514059

[4] Allsopp R (2001) Options for vector control against trypanosomiasis in Africa. Trends Parasitol 17: 15–19. 11137735 10.1016/s1471-4922(00)01828-6

[5] Gibson G, Torr SJ (1999) Visual and olfactory responses of haematophagous Diptera to host stimuli. Med Vet Entomol 13: 2–23. 10194745 10.1046/j.1365-2915.1999.00163.x

[6] Omolo MO, Hassanali A, Mpiana S, Esterhuizen J, Lindh J, et al (2009) Prospects for developing odour baits to control Glossina fuscipes spp., the major vector of human African trypanosomiasis. PLoS Negl Trop Dis 3: e435. 19434232 10.1371/journal.pntd.0000435 PMC2674566

[7] Den Otter CJ (1991) Olfactory responses of tsetse flies to phenols from buffalo urine. Physiol Entomol 16: 401–410.

[8] Den Otter CJ, Van der Goes van Naters WM (1993) Responses of individual olfactory cells of tsetse flies (*Glossina m. morsitans*) to phenols from cattle urine. Physiol Entomol 18: 43–49.

[9] Saini RK, Hassanali A, Andoke J, Ahuya P, Ouma WP (1996) Identification of major components of larviposition pheromone from larvae of tsetse flies *Glossina morsitans morsitans* Westwood and Glossina morsitans centralis Machado. J Chem Ecol 22: 1211–1220. 24226080 10.1007/BF02266961

[10] BOGNER F (1992) Response properties of C02-sensitive receptors in tsetse flies (Diptera: *Glossina palpalis*). Physiol Entomol 17: 19–24.

[11] Vale GA (1980) Field studies of the response of tsetse flies (Glossinidae) and other Diptera to carbon dioxide, acetone and other chemicals. Bull Entomol Res 70: 563–570.

[12] Vale GA, Hall DR (1985b) The use of 1-octen-3-ol, acetone and carbon dioxide to improve baits for tsetse flies, *Glossina spp.* (Diptera: Glossinidae), to host odour. Bull Entomol Res 75: 219–231.

[13] Syed Z, Guerin PM (2004) Tsetse flies are attracted to the invasive plant *Lantana camara* . Journal of insect physiology 50: 43–50. 15037092 10.1016/j.jinsphys.2003.09.007

[14] Saini RK, Hassanali A (2007) A 4-alkyl-substituted analogue of guaiacol shows greater repellency to savannah tsetse (*Glossina spp.*). J Chem Ecol logy 33: 985–995. 10.1007/s10886-007-9272-7 17404820

[15] Willemse L, Takken W (1994) Odor-induced host location in tsetse flies (Diptera: Glossinidae). J Med Entomol 31: 775–794. 7815390 10.1093/jmedent/31.6.775

[16] Lehane M (2005) The Biology of Blood-Sucking in Insects. Second. Cambridge, UK: CUP.

[17] Gikonyo NK, Hassanali A, Njagi PG, Gitu PM MJ (2002) Odor composition of preferred (buffalo and ox) and non-preferred (waterbuck) hosts of some Savanna tsetse flies. J Chem Ecol 28: 969–981. 12049234 10.1023/a:1015205716921

[18] Gikonyo NK, Hassanali A, Njagi PGN, Saini RK (2003) Responses of *Glossina morsitans morsitans* to blends of electroantennographically active compounds in the odors of its preferred (buffalo and ox) and non preferred (waterbuck) hosts. J Chem Ecol 29: 2331–2345. 14682515 10.1023/a:1026230615877

[19] Mwangi MT, Gikonyo NK, Ndiege IO (2008) Repellent properties of delta octalactone against the tsetse fly *Glossina morsitans morsitans* . J Insect Sci 8: 7–10. 20298116 10.1673/031.008.4301 PMC3127398

[20] Vale GA (1985a) Flight as a factor in the host-finding behaviour of tsetse flies (Diptera: Glossinidae). Bull Entomol Res 70: 299–307.

[21] Vale GA, Hall DR, Gough AJE (1988) The olfactory responses of tsetse flies, *Glossina spp.* (Diptera: Glossinidae), to phenols and urine in the field. Bull Entomol Res 78: 293–300.

[22] Mireji PO, Mabveni AM, Dube BN, Ogembo JG, Matoka CM, Mangawiro TNC (2003) Field responses of tsetse flies (Glossinidae) and other Diptera to oils in formulations of deltamethrin. Int J Trop Insect Sci 23: 317–323.

[23] Saini RK, Hassanali A, Andoke J, Ahuya P, Ouma WP (1996) Identification of major components of larviposition pheromone from larvae of tsetse flies *Glossina morsitans morsitans* Westwood and *Glossina morsitans centralis* Machado. J Chem Ecol 22: 1211–1220 doi:10.1007/BF02266961 24226080 10.1007/BF02266961

[24] Den Otter CJ, Saini RK (1985) Pheromone perception in the tsetse fly. Entomol Exp Appl 39: 155–161.

[25] Clyne PJ, Warr CG, Freeman MR, Lessing D, Kim J, Carlson JR (1999) A novel family of divergent seven-membrane proteins: candidate odorant receptors in *Drosophila* . Neuron 22: 327–338. 10069338 10.1016/s0896-6273(00)81093-4

[26] Vosshal LB, Amrein H, Morozow PS, Rzhetsky A, Axel A (1999) A spatial map of olfactory receptor expression in the *Drosophila* antenna. Cell 96: 725–736. 10089887 10.1016/s0092-8674(00)80582-6

[27] Clyne PJ, Warr CG, Carlson JR (2000) Candidate taste receptors in *Drosophila* . *Science* 287: 1830–1834. 10710312 10.1126/science.287.5459.1830

[28] Ghaninia M, Ignell R, Hansson BS (2007) Functional classification and central nervous projections of olfactory receptor neurons housed in antennal trichoid sensilla of female yellow fever mosquitoes, *Aedes aegypti* . Eur J Neurosci 26: 1611–1623. 17880395 10.1111/j.1460-9568.2007.05786.x PMC2121139

[29] Robertson HM, Warr CG, Carlson JR (2003) Molecular evolution of the insect chemoreceptor gene superfamily in *Drosophila melanogaster* . Proc Natl Acad Sci USA 100: 14537–14542. 14608037 10.1073/pnas.2335847100 PMC304115

[30] Fuss W (2009) Does life originate from a single molecule? Chirality 21: 299–304. 18537164 10.1002/chir.20576

[31] Zwiebel LJ, Takken W (2004) Olfactory regulation of mosquito-host interactions. Insect Biochem Mol Biol 34: 645–652. 15242705 10.1016/j.ibmb.2004.03.017 PMC3100215

[32] Fuss SH, Ray A (2009) Mechanisms of odorant receptor gene choice in Drosophila and vertebrates. Mol Cell Neurosci 41: 101–112. 19303443 10.1016/j.mcn.2009.02.014

[33] de Bruyne M, Baker TC (2008) Odor detection in insects: volatile codes. J Chem Ecol 34: 882–897. 18535862 10.1007/s10886-008-9485-4

[34] Nozawa M, Nei M (2007) Evolutionary dynamics of olfactory receptor genes in Drosophila species. Proc Natl Acad Sci USA 104: 7122–7127. 17438280 10.1073/pnas.0702133104 PMC1855360

[35] Krieger J, Raming K, Dewer YM, Bette S, Conzelmann S, et al (2002) A divergent gene family encoding candidate olfactory receptors of the moth *Heliothis virescens* . Eur J Neurosci 16: 619–628. 12270037 10.1046/j.1460-9568.2002.02109.x

[36] Jones WD, Nguyen TA, Kloss B, Lee KJ, Vosshall LB (2005) Functional conservation of an insect odorant receptor gene across 250 million years of evolution. Curr Biol 15: R119–R121. 10.1016/j.cub.2005.02.007 15723778

[37] Vosshall TN, Vosshall LB (2009) Mechanisms in the Insect Olfactory System. Curr Opin Neurobiol 19: 284–292 doi:10.1016/j.conb.2009.07.015 19660933 10.1016/j.conb.2009.07.015 PMC2752668

[38] Hansson BS, Stensmyr MC (2011) Review: Evolution of Insect Olfaction. Neuron 72: 698–711. 22153368 10.1016/j.neuron.2011.11.003

[39] Pellegrino M, Nakagawa T (2009) Smelling the difference: controversial ideas in insect olfaction. J Exp Biol 212: 1973–1979. 19525421 10.1242/jeb.023036 PMC2702451

[40] Montell C (2010) A Taste of the Drosophila Gustatory Receptors. Curr Opin Neurobiol 19: 345–353 doi:10.1016/j.conb.2009.07.001.A 10.1016/j.conb.2009.07.001 PMC2747619 19660932

[41] Robertson HM, Wanner KW (2006) The chemoreceptor superfamily in the honey bee, *Apis mellifera*: expansion of the odorant, but not gustatory, receptor family. Genome Res 16: 1395–1403. 17065611 10.1101/gr.5057506 PMC1626641

[42] Sánchez-Gracia A, Vieira FG, Rozas J (2009) Molecular evolution of the major chemosensory gene families in insects. Heredity 103: 208–216. 19436326 10.1038/hdy.2009.55

[43] Fiala A (2007) Olfaction and olfactory learning in Drosophila: recent progress. Curr Opin Neurobiol 17: 720–726 doi:10.1016/j.conb.2007.11.009 18242976 10.1016/j.conb.2007.11.009

[44] McBride CS, Arguello JR, O'Meara BC (2007) Five Drosophila genomes reveal nonneutral evolution and the signature of host specialization in the chemoreceptor superfamily. Genetics 177: 1395–1416. 18039874 10.1534/genetics.107.078683 PMC2147975

[45] Gardiner A, Barker D, Butlin RK, Jordan WC, Ritchie MG (2008) Drosophila chemoreceptor gene evolution: selection, specialization and genome size. Mol Ecol 17: 1648–1657. 18371013 10.1111/j.1365-294X.2008.03713.x

[46] Parra G, Bradnam K, Korf I (2007) CEGMA: a pipeline to accurately annotate core genes in eukaryotic genomes. Bioinformatics (Oxford, England) 23: 1061–1067. 10.1093/bioinformatics/btm071 17332020

[47] McQuilton P, Susan E, Pierre St, Thurmond J (2012) FlyBase Consortium FlyBase 101 – the basics of navigating FlyBase. Nucleic Acids Res 40: D706–14 doi:10.1093/nar/gkr1030 22127867 10.1093/nar/gkr1030 PMC3245098

[48] Megy K, Emrich SJ, Lawson D, Campbell D, Dialynas E, et al (2012) VectorBase: improvements to a bioinformatics resource for invertebrate vector genomics. Nucleic Acids Res 40: 1–6. 22135296 10.1093/nar/gkr1089 PMC3245112

[49] Altschul SF, Lipman DJ (1990) Protein database searches for multiple alignments. Proc Natl Acad Sci USA 87: 5509–5513. 2196570 10.1073/pnas.87.14.5509 PMC54354

[50] Benoit JB, Geoffrey MA, Veronika M, Tyler BK, Jana B, Zhang Qirui, et al (2013) A novel highly divergent protein family from a viviparous insect identified by RNA-seq analysis: a potential target for tsetse fly-specific abortifacients. PLOS Genetics *(in Press)*. 10.1371/journal.pgen.1003874 PMC3998918 24763277

[51] Carver T, Berriman M, Tivey A, Patel C, Bohme U, et al (2008) Artemis and ACT: viewing, annotating and comparing sequences stored in a relational database. Bioinformatics 24: 2672–2676. 18845581 10.1093/bioinformatics/btn529 PMC2606163

[52] Fox AN, Pitts RJ, Robertson HM, Carlson JR, Zwiebel LJ (2001) Candidate odorant receptors from the malaria vector mosquito *Anopheles gambiae* and evidence of down-regulation in response to blood feeding. Proc Natl Acad Sci USA 98: 14693–14697. 11724964 10.1073/pnas.261432998 PMC64743

[53] Boratyn GM, Schaffer A A, Agarwala R, Altschul SF, Lipman DJ, et al (2012) Domain enhanced lookup time accelerated BLAST. Biology Direct 7: 12. 22510480 10.1186/1745-6150-7-12 PMC3438057

[54] Marchler-Bauer ALS, Anderson JB, Chitsaz F, Derbyshire MK, Deweese-Scott C, et al (2011) CDD: a Conserved Domain Database for the functional annotation of proteins. Nucleic Acids Res 39: D225–229. 21109532 10.1093/nar/gkq1189 PMC3013737

[55] Krogh A, Larsson B, von Heijne G, Sonnhammer EL (2001) Predicting transmembrane protein topology with a hidden Markov model: application to complete genomes. J Mol Biol 305: 567–580. 11152613 10.1006/jmbi.2000.4315

[56] Conesa A, Götz S, García-Gómez JM, Terol J, Talón M, et al (2005) Blast2GO: a universal tool for annotation, visualization and analysis in functional genomics research. Bioinformatics (Oxford, England) 21: 3674–3676. 10.1093/bioinformatics/bti610 16081474

[57] The UniProt Consortium (2013) Update on activities at the Universal Protein Resource UniProt in 2013. Nucleic Acids Res 41: D43–D47. 23161681 10.1093/nar/gks1068 PMC3531094

[58] Edgar Robert C (2004) MUSCLE: multiple sequence alignment with high accuracy and high throughput,. Nucleic Acids Res 32 (5) 1792–97. 15034147 10.1093/nar/gkh340 PMC390337

[59] Waterhouse AM, Procter JB, Martin DM, Clamp M, Barton GJ (2009) Jalview Version 2–a multiple sequence alignment editor and analysis workbench. Bioinformatics 25: 1189–1191. 19151095 10.1093/bioinformatics/btp033 PMC2672624

[60] Cole C, Barber JD, Barton GJ (2008) The Jpred 3 secondary structure prediction server. Nucleic acids res 36: W197–W201. 18463136 10.1093/nar/gkn238 PMC2447793

[61] Wheelan S, Goldman N (2001) A general empirical model of protein evolution derived from multiple protein families using a maximum-likelihood approach. Mol Biol Evol 18 (5) 691–9. 11319253 10.1093/oxfordjournals.molbev.a003851

[62] Tamura K, Peterson D, Peterson N, Stecher G, Nei M, et al (2011) MEGA5: molecular evolutionary genetics analysis using maximum likelihood, evolutionary distance, and maximum parsimony methods. Mol Biol Evol 28: 2731–2739. 21546353 10.1093/molbev/msr121 PMC3203626

[63] Mortazavi A, Williams BA, Mccue K, Schaeffer L, Wold B (2008) Mapping and quantifying mammalian transcriptomes by RNA-Seq. Nat Methods 5: 1–8. 18516045 10.1038/nmeth.1226 PMC13303166

[64] Guo S, Kim J (2007) Molecular evolution of Drosophila odorant receptor genes. Mol Biol Evol 24 (5) 1198–207 doi:10.1093/molbev/msm038 17331958 10.1093/molbev/msm038

[65] Benton R (2006) On the ORigin of smell: odorant receptors in insects. Cell Mol Life Sci 63: 1579–1585. 16786219 10.1007/s00018-006-6130-7 PMC11136275

[66] Pitts RJ, Fox AN, Zwiebel LJ (2004) A highly conserved candidate chemoreceptor expressed in both olfactory and gustatory tissues in the malaria vector *Anopheles gambiae* . Proc Natl Acad Sci USA 101: 5058–5063. 15037749 10.1073/pnas.0308146101 PMC387373

[67] Bouyer J, Pruvot M, Bengaly Z, Guerin PM, Lancelot R (2007) Learning influences host choice in tsetse. Biology letters 3: 113–116 doi:10.1098/rsbl.2006.0578 17251119 10.1098/rsbl.2006.0578 PMC2375919

[68] Hargrove JW (1976) Amino acid metabolism during flight in tsetse flies. J Insect Physiol 22 (2) 309–313. 1249439 10.1016/0022-1910(76)90040-8

[69] Bellmann D, Richardt A, Freyberger R, Nuwal N, Schwärzel M, et al (2010) Optogenetically Induced Olfactory Stimulation in Drosophila Larvae Reveals the Neuronal Basis of Odor-Aversion behavior. Front Behav Neurosci 4 (June) 27 doi:10.3389/fnbeh.2010.00027 20577637 10.3389/fnbeh.2010.00027 PMC2889724

[70] Marinotti O, Calvo E, Nguyen QK, Dissanayake S, Ribeiro JM, et al (2006) Genome-wide analysis of gene expression in adult *Anopheles gambiae* . Insect Mol Biol 15: 1–12. 16469063 10.1111/j.1365-2583.2006.00610.x

[71] Parisi M, Nuttall R, Edwards P, Minor J, Naiman D, et al (2004) A survey of ovary-, testis-, and soma-biased gene expression in *Drosophila melanogaster* adults. Genome Biol 5: R40. 15186491 10.1186/gb-2004-5-6-r40 PMC463073

[72] McGraw LA, Clark AG, Wolfner MF (2008) Post-mating gene expression profiles of female *Drosophila melanogaster* in response to time and to four male accessory gland proteins. Genetics 179: 1395–1408. 18562649 10.1534/genetics.108.086934 PMC2475742

[73] Bionaz M, Periasamy K, Rodriguez-Zas SL, Everts RE, Lewin HA, et al (2012) Old and new stories: revelations from functional analysis of the bovine mammary transcriptome during the lactation cycle. PLoS ONE 7: e33268. 22428004 10.1371/journal.pone.0033268 PMC3299771

[74] Bohbot J, Pitts RJ, Kwon HW, Rutzler M, Robertson HM, et al (2007) Molecular characterization of the *Aedes aegypti* odorant receptor gene family. Insect Mol Biol 16: 525–537. 17635615 10.1111/j.1365-2583.2007.00748.x PMC3100214

[75] Zhou X, Slone JD, Rokas A, Berger SL, Liebig J, et al (2012) Phylogenetic and Transcriptomic Analysis of Chemosensory Receptors in a Pair of Divergent Ant Species Reveals Sex-Specific Signatures of Odor Coding. PLOS Genet 8 (8) e1002930. 22952454 10.1371/journal.pgen.1002930 PMC3431598

[76] Hill CA, Fox AN, Pitts RJ, Kent LB, Tan PL, et al (2002) G Protein-Coupled Receptors in *Anopheles gambiae* . Science 298: 176–178. 12364795 10.1126/science.1076196

[77] Kent LB, Walden KKO, Robertson HM (2008) The Gr Family of Candidate Gustatory and Olfactory Receptors in the Yellow-Fever Mosquito *Aedes aegypti* . Chem Senses 33: 79–93. 17928357 10.1093/chemse/bjm067

[78] Richards S, Gibbs R, Weinstock G, Brown S, Danell R, et al (2008) The genome of the model beetle and pest *Tribolium casteneum* . Nature 452 (7190) 949–955. 18362917 10.1038/nature06784

[79] Engsontia P, Sanderson AP, Cobb M, Walden KKO, Robertson HM, et al (2008) The red flour beetle's large nose: An expanded odorant receptor gene family in *Tribolium casteneum* . Insect Biochemistry and Molecular Biology 38: 387–397. 18342245 10.1016/j.ibmb.2007.10.005

[80] Robertson HM, Gadau J, Wanner KW (2010) The insect chemoreceptor superfamily of the parasitoid jewel wasp *Nasonia vitripennis* . Insect Mol Biol 19 (1) 121–136. 10.1111/j.1365-2583.2009.00979.x 20167023

