# Supplementary material for: Odorant and Gustatory Receptors in the Tsetse Fly Glossina morsitans morsitans
Source: PLoS Negl Trop Dis. 2014 Apr 24;8(4):e2663. doi: 10.1371/journal.pntd.0002663 (PMC3998910; doi:10.1371/journal.pntd.0002663)
Supplement: Dataset S1 — Annotated amino acid sequences of Glossina morsitans morsitans ORs and GRs. Each sequence name is followed by annotation identity (previous temporary identity), scaffold number and the coordinates within the scaffold. (PDF) [file pntd.0002663.s001.pdf]

Supplementary material
Dataset S1. Annotated amino acid sequences of Glossina morsitans morsitans ORs and GRs.

>GmmOR1|Orco GMOY005610 (TMP007718) scf-648683 14760:27560 reverse
MFKQTMANDLQPGKYVGLVADLMPNIKLMKFSGLFMHAFTSGSAVGKKIYSSIHLALILL
QFLSILINMALNADEVNELSGNTITVLFFTHSITKFIYLACSQKNFYRTISIWNQVNTHP
LFAESDARYHSIALAKMRKLFFLVMLTTVTSAIAWITLTFFGESVKFAVDKENNSTMTVE
IPRLPIKSFYPWDASSGISYIVSFIYQAYFILFALSHANLCDVLFCSWLIFACEQLQHLK
SIMKPLMELSASLDTFRPDSGALFRSLSAHSKAELIENEEKEPPPSNGLDLSGIYSSKAD
WGAQFRAPSTLQTFNNNNPNGLTKKQEMMVRSAIKYWVERHKHVVRLVAAIGDTYGVALL
LHMLTATIKLTLLAYQATKITGVNVYALGVVGYLGYSLAQVFHFCIFGNRLIEESSSVME
AAYSCHWYDGSEEAKTFVQIVCQQCQKAMSISGAKFFTVSLDLFASQWSHISWCWCNLNE
VIASFAIKFSSSSSLRPSSLITKRNHGECSKISYLVVYLYFV
>GmmOR2 GMOY005796 (TMP007907) scf-648756 29714:32260 reverse
MEYRAHLETNTAFRYHWLVWQLTGIIQPRYFSTSLYRLYTVLVNILVTFLFPLTFVINVF
FSKNLQQLCENLIITLCDITANLKFLNVFLVRKELQQIKIILECLDKRLNTEEEYRQLKR
AIRTAQLSFCIFLVISTTGTTLSLFMVLYSEERSLLFPAWYGLDWQKNDLAYAVCFTYQL
VALVVQAIQNVANDSYPPAYLIILTSHMRNLELRVRSVGLSPANNKENMFLSEKEQQLDF
KKFNDCIEDFIQINRLYDLIQKILSKACLAQFICTALVQCVVGLHILYLLDESDDYGAQI
LSFIFFLAVTMEVFIICYFGHYMSAQSLALIDAFYECGWIPQTLAFRRNLIITLMRTQRY
AILYAGSYIPVDLPTFLLLMKYAYSTFTLLLRFK
>GmmOR3 GMOY004772 (TMP006862) scf-648228 109437:110684 reverse
MVVTKIHTWKAFEYHWRLWTFFGLKPPPRNSVWFKPYVAYAILLNISVTFFFPCTLIINL
ILAKNMNEVCENLYHTITDVVCNIKFLNMFLVRKKLLQINQILKRLDMRAQTLEEITELQ
RGVNSARNCFKIVGRFFCVALITSQLVAYLSPERILMYPAWFPWDWRASKMNFLYAHSYQ
LYGLTLQTAQNLGSDTYPQAYLVVLIGHIKALSLRIKALGSEVTPFSTSDDEFKKKNENH
LYRELVDCIIDHETINDLFRIVQQGISSTCLAQFFCTGLAQCTMGVYTFYVGLDYSKLPN
LIAYFSAVTAEIFILCYYGDLFCQANEKLINSIYSCNWVDPHRFLLQRSQKINTVMAGNI
IPVSLSTFVTVKTESYRLHLHFKSHIM
>GmmOR4 TMP_Or4 scf-642438 111181:112437 forward
MFLDTLQFFRNNWLSWRLLGIVLPKGEQNHKLIKYLLWSTIVNVVATFLFPLHLLLGIFQ
EQPQSTRFESVAICVTSIATSLKFIIYARKLQHVKQMGKLFQRLDARISNDNERQFYQKH
IRRNVIRIQTMFIVVYISVGITVTVAFIFSQERRLFYPGWLPFDWHRSIKYYMAALGFQL
ISIFFQILQNFANDCFTPKALCLLSGHIELLYMRVANIGYVNRVAYQQNRNLRELNECVL
DQKHLYQLFDVIQSIISWPMFLQFLASTVNMCMAMVTLLFFVTDILERIYYVMYFAAMCL
QIFPTCYYGSDFEIKFERLHYAVFSSNWTEGTQCFKRHMMLFTERALRETRAMAGGVFRI
HLDTFFATIKGAYSLFAVVITMKN
>GmmOR5a GMOY012018-PA (TMP002414) scf-639717 1: 6297 MW: 51399.66
SELDKRVSTADECDYFIRQHQRKANSAIKIFFFVYLGTNSFAFMGAIVDQRLMAPAWFPF
DWKSSSTLYWSALLYQFIGLNMLIMQNLVNDTVGPMSLCLLSGHVHLLAMRVARIGYNKR
KSQKYHEDELKLCIEDHQKLLKAFTLLESSMSWLQFILFFTSGLNTCLGVVNFLFYSRIL
YDYIYYGCFLLALGVEVFPCYFYGSVLLEEFKHLPYAIFSSNWVEQSRNYRQNTTIFLEM
ALKSVTMLAGGIVETNLDSFFAIYKAAYSLFTVILTMKIGYNKRKSQKYHEDELKLCIED
HQKLLKAFTLLESSMSWLQFILFFTGGLNTCLGVVNFLFYSRILYDYIYYGCFLLAIGME
VFPCYFYGSVLLEEFKHLPYAIFSSNWVEQSRNYRQNTTIFLEMALKSVTMLAGGIVEIN
LDSFFAIYKAAYSLFTVILTMK
>GmmOr5b GMOY012018-PA (TMP002414) scf-639717 - 1: 6297 MW: 35691.12
SELDKRVSTADECDYFIRQHQRKANSAIKIFFFVYLGTNSFAFMGAIVDQRLMAPAWFPF

DWKSSSTLYWSALLYQFIGLNMLIMQNLVNDTVGPMSLCLLSGHVHLLAMRVARIGYNKR
KSQKYHEDELKLCIEDHQKLLKIGYNKRKSQKYHEDELKLCIEDHQKLLKAFTLLESSMS
WLQFILFFTGGLNTCLGVVNFLFYSRILYDYIYYGCFLLAIGMEVFPCYFYGSVLLEEFK
HLPYAIFSSNWVEQSRNYRQNTTIFLEMALKSVTMLAGGIVEINLDSFFAIYKAAYSLFT
VILTMK
>GmmOr5c GMOY012018-PC (TMP002414) scf-639717 - 5069: 6297 MW: 33056.258
MKIEWWSKLDSTVAYGRFWSCFRILGVADFRYKALSQFYVICLTLFVTVYCPIHLLIGLL
MLTDAGDFFKNFSMTLTSLVCSLKYFFLRLNLNKIHQLIKIYSELDKRVSTADECDYLIG
YNKRKSQKYHEDELKLCIEDHQKLLKAFTLLESSMSWLQFILFFTGGLNTCLGVVNFLFY
SRILYDYIYYGCFLLAIGMEVFPCYFYGSVLLEEFKHLPYAIFSSNWVEQSRNYRQNTTI
FLEMALKSVTMLAGGIVEINLDSFFAIYKAAYSLFTVILTMK
>GmmOr5d |GMOY012018-PD (TMP002414) scf-639717 - 1: 902 MW: 31461.035
SELDKRVSTADECDYFIRQHQRKANSAIKIFFFVYLGTNSFAFMGAIVDQRLMAPAWFPF
DWKSSSTLYWSALLYQFIGLNMLIMQNLVNDTVGPMSLCLLSGHVHLLAMRVARIGYNKR
KSQKYHEDELKLCIEDHQKLLNSMSWLQFILFFTSGLNTCLGVVNFLFYSRILYDYIYYG
CFLLLALGVEVFPCYFYGSVLLEEFKHLPYAIFSSNWVEQSRNYRQNTTIFLEMALKSVTM
LAGGIVETNLDSFFAIYKAAYSLFTVILTMKIGYNKRKSQKYHEDELKLCIEDHQKLLKY
LKYSYI
>GmmOR6 GMOY009475 (TMP011670) scf-651846 662444:668878 reverse
MLLALSRGVGVKTQSSQASEYLFNVINFLAQDFHRKWTFGFFFQSFVANGTVVLFMPILF
NLSYLNDSMQFDLGQLFTSVQAAINICAIPIKFITMQLYMKNLHRINEMLNILDGRCKHP
EEFSKLRHCAITGNRIYVGAIIVYFSYSISTCMGFMLTGQAAYNIYIPGINWRNSLFEFV
IQGLVEFASMNLICLHQTVYDSYSGIYLYIIRIHIQILNERVKRLGSNLKNNEKQSYEEL
IQSILDHKLILREGMALKSSRDNQYPLVAAISPMISLTIFVQFAITAAMLATTMINISFF
SNVVGRIASIFYIILVFAQSSLCCYQATCLVSDADELPVSIFHCQWVEQGYRFRKMIIYF
MVNTQKPITFTAGKLFPVNMASVLSNY
>GmmOR7 TMP_Or7-RA scf-651846 655714:658243 reverse
MESVIPNTVEDQQSGERHSINKGRFDSQLNINYTNNVTNAFDRNARTMHGTMFLFNGFRF
LGVYMPEKNRLFYLIWSFLINSTVTVYLPVAFILSFVKISGDDLQIGNLLTSAQVAINVV
GCSVKIILMGFLLPKLLSTEKFLNRLDERCRTAEELASINKFTKQGNRFVVLFSVAYWSY
STSTCISAVAFGRLPYNIYNPFFDHHQSIGHFILSVLMEMTLVNIACFQQVVDDSYAVIY
VNILRTHVDLLLKRIKRLGLTVSMSHEQTYEELKLCIIDHKYIIQFTITATILGTTLINI
LLFATNFASIVASCFYVLAVVVEIFPLCYYTQYLMDESNLLAEVVFHSNWMEQNLPYQKM
LIFFMQRSQRVMEFTAGKLFPITLNSFLSVSES
>GmmOR8 TMP_Or8-RA scf-651846 - 655986:662305 reverse
MLVDTVADYKSPISSFEATTYLFRIYRVLGINKPQRHKNLYIFYTIILNGVVNFLQPIAF
TMNYFTNELVSKLGSLLTSIEIVINVYGCTAKFIIIAWLLPRSNATVQLLKKLDERCQAP
DELELLKSIKKFGRLVVVVLAVSYLSYSVSQFICSLIAGHPPYGLYIPYINWKRSFWEFL
VASSIEFLLMNVSCIVQASNDAYPIIYINILRTHIKILLKRLNRLGTGADKSQDEHLEEL
KLCIKDHKNLNRFIAAFSLFETIAPIISTTVFVQFMITAAIIALTLINMLFFTTNISAVA
NSCVYIADLVLEIFPICYYANCLIDDNDLLSMEIFHSAWPEQNKIYRKMLIVFMQRSQQS
MSLTAGKMFPINLNTFINLQYRFIYNFYNNICAIYNNCHHIGYDSY
>GmmOR9 TMP_Or9-RA scf-652157 136730:138889 reverse
MLTFRKFFKMAHFRGDGPPPKTRDAVLYLFRGITIIGLLTPASNKKCFYVWSLFINSLVT
LYMPVGFLLSFIMRLSSFSPSEFFTSLQISINCVGASLKLFVFSLMYKRLIKATKYMDRL
DKRPIDEQGEFTLRQAVAFSNCSSLLFTLLYLSYSSSTFLSSVINRRPPYQIYNPVIEWR
NSTRNFIIQSAIEYVMIDVHCYQQALLDAYPVIYIMIIRAHLHILSRRISQLGKDDKLKK
NQRYEELVQCVLDHKNIMQLYNLFSPVISGTMFVQFLIIGIILGVTTIHIVLFANFFAIV
ASLFYVASILAETFPCSYLANSLMDDSDELSLAIFHSGWFNEEPRYKQVVVFFLQHTQKT

LVLTAMKIFPITMNSNINVVVKFAFSVYTLISQMDLRQKLKDSVAGQEA
>GmmOR10 TMP_Or10 scf-644232 131824:133273 forward
MDGREHCRYLKTFYKHQCWVFRLFGLWKLSADMTRSHQLLHALYFNYILTIWVLSLDASC
IMQLVIKAGDLNEVIEVFSIFATALAVLAKFLSIKMKNHLYRQWCEIVESSTFRPNNERE
IQLFQQSERLARLIRNAYCVLSLIALNMLIFIVDDRGLPLSVYSPFDMNRKWGYLSTYGY
QYIAASICCYLNIAFDSLSASFLIHLKGQMDILCDRLKNFGKYSECNNDHKITEELKNCI
KYYEEILKVAHLIEDLISLPISIQIICSVLVMVANFFGMAFLAEPGDYAYFFKMLIYQLC
MLSQIYILCYFPNEVTDRSQTISRSLYSAEWFSWSQMNRKLTLLMMDRFDVPIRFRSIIP
TYSFNLAAFTSVFTTKRQQNILLCFIKKHEQLKYFSANLKLKRNLQRTTKSLIGPKSNN
RETSTADVKERNSWTELDELDEYS
>GmmOR11 GMOY0110761 (TMP012981) scf-652156 1267206:1269387 reverse
MDNSAESVKMLYSKQILIFRLVSIWDLAANAGYYKRLAFAVYFWIVAIFLVALFALLLII
QIFCDINNISEVIRVMFNLASSLTVLGKFLTVKMKNRSFQQLFDLLHSTEYLPRDLKEDK
LFKRALQLSKTVLKVYGGMSLISINTTFLIQFAKDTTELPLPIYEPIDATVPWKYFIMYF
YEYLGFGLCCVMNIAYDSLGAAFFIHIKGQVDILSKRLEEIGHKSSSKQNHSHINEELKA
CAIYYDRILELTHMMEDLMCLPLSIQILFTLTSEKLSLAIYRAGWVDWNEKNRKLALQMM
GRLDLPIRIKTINRCYSFNLAAFTSIVNSSYSYFALLKNFN
>GmmOR12 GMOY009271 (TMP011461) scf-651831 454103:455258 reverse
MLHVPITFTFTTLMWLKVIFSSNLNDVTDVLHMAFTETGLVIKILSAWRFARLLQFVFLE
WQTNNLFSLKSANEQLIWNDQFKSFKITAFVYMSCSLSVVIFSFISVPLMKTYRLPFAFW
TPAGWEQSSYFWYICFYDFIGITFTCISNCTVDMYFCYLLKHITVCLRMISVRLVKLGYS
SEDVTKNLKEIITFHNKLKRMSRHCEKVISYPILGQILFSSMVLCFSIYRLQAISFIETP
FDFLSVFQYMWVMAAQIYLPCHYCNELTLQSGALHTAIYNCNWIAMTAAERKTILLFMLY
IKRPIILKAGHFFEIGLPIFTKTMNNAYSLLALVLNMSDS
>GmmOR13 GMOY003312 (TMP005369) scf-645812 24048:26362 reverse
MIDLFERQRFCLRVMGHNFVRDRSQQSRRWHNTRCTYKYILCLFLVVSAQVPMINYTIYY
LDHVELATASLSICFTNILTTVKIITFLLYKWEFAALMEKMEEMYYEVRKAETKAKLKRD
NDYAGDLTRMYWNSCCCTGAYFMTTPILKIIWSKLQGMDVPLELPMPMRFSFDFESTPGY
EFCYIYTGLTTLVVIAYAVAIDGLFIGFTINLKAHFIALQSIIETNNFLKSESELQRDLS
ACIGYHNKILSLAFKLRDIYRPIIFAQFLMTSLQVCVIVYQMVTVSASQYINAQHIFLKN
CLFLCSILLQLFIYCYGGEILKLESLRVGVSVEISHWYNLKPSHRRILCLLMLRSQREAI
IKAGFYEASLANFTAILKAAMSYITLIQSIE
>GmmOR14 GMOY001365 (TMP003388) scf-641298 67504:69789 forward
MFFKQWLPKADDALPSVNGLSRHFTVQQYTFAAIGLDPKSLQRPIFNKMLALVPMLGLFS
LVIPMIGYASLYKSDILKVTDALSPVWEGLLALAKFFYFIWNRQKVIQLLRKIWIKNLEV
SSNPEELTIIAEANHRDYLFSLTFCINVITTGVLALAAPLIIATFYTLQGEKFLNVLEPP
LKATYPFDFHTPNGLIALYVWDSLFVYFIIFGNLSIDGIFSWFTCNIAAHFRILRLRLKY
AGQENGGNITKTTVNGCIDLHRQTIELAEEFNKIFRVNVFIKFTISCLQIACLAFQLVRG
KEKVDQIFHFSFLTSVTLQFLLYCYGGQKIKDEVIMSNYGL
>GmmOR15 TMP_Or15-RA scf-651027 85495:88783 forward
MLLHTLQLKRYFWVTRRSFNLIGIDITALDYHDIVKYPMRCFLFTAFAAVLAWAMTIHVY
EYRDDFGEFADTSGMLLQTIIALWKTLVFLFKRKEICDLMQNVWQCNINVNPQEFHIILK
FNSQNFTISALYMVLVSSTVFGSFLVPLIYMFEFYRKNGEKIWLPPQKGGYFWDYSNAIG
YSVLYICHLLGIFFVAAFSIGVDTLCPWLVSNIVVQYHVMYYRLRDIAELSYEISTEKLN
AKIIECVKCHRQVLNLSNQLENFFAEIIFIKFVISGLLICSLAFRLVRAEGQFYILLYQL
VFLTTVSTQLLMYCYSGQRLKNESSQVASEIYSIFEWSHLSRNSQKLLLFAMMRSQRECH
LTGAFFMVDLSLFVWVRERERDVCMYVKQKKKKKKRTNIFNFWFCLKKKSYHFVLLLHVF
LCYLLKVLRTGGSLIAMLKTLDEKQV
>GmmOR16 TMP_Or16-RA scf-649095 5203:6553 reverse

MPDKKCAKFLSIQQRNLAVLGFDLNAAERRYLVEKPLKLLFVLSCNFYWTYGLMNFAIYN
IENFDEITGSLSVFNQDILLFFKMFIFFAKADKYLNLIKSMNKLADKAKNQEYDEWMSEN
RLAELIAKVYSYTCRVAVAFSATVPLIYSVYVTYTAGELKLKLPVKARFTLDGKLSYFAF
NYITFVIHLNSMASLTVGLDSLYFWFIYNISAHFRILRKKLEIMAVNLKTNLQYSIQDDL
GAIVVYHLEVIRFIKSMNEIFGEILWAEVTMSCLQMCFATHALMSDVDVSNAPFNIVVVF
AVIIQLAIYCFGGEKIREESISLCSDVYLLFPWHKMSMHQRRLMLLPLLRAQKEAYLKGL
FFQVDRNLFVFVSKRIRSVTLIPVGFA
>GmmOR17 GMOY005386 (TMP007492) scf-648564 107291:115929 forward
MSFRLGNVSFNLTRKIIVAYGLIETHLFKYHESNIPKICLNDLIRAITIHEMDYKFKKLE
PHNYIKNGSDELNHIIVWLMKAYSDNILNSVETGLKDKCLEMILVATMWKIKQSLMNHFP
SDPRKGQIGSIELNLWLAQMSGVSVMRLRQNSSDINIFIFLYTALITILVTFVYTACEFY
DLALNWYDLNILTQNSCISLTHLAGLAKIINALYKLKDIQKVLDKLKYGLRTYTISKEQR
QTFLDGELENKLLLSVYVAIVATTGLLGMFVLFLSIQVLLHPVKMAGKTFPYRAVVPSWV
PFPLQLLYMSLSVLIFAMQIVAIDYLNINLLNQLRYQLNILNLAFDKLSFEKEQKNPAPH
SRRLNAIIEHHCLLKEVCQDIESIFSVSILLQFFTSLIIFAMTGFQATVQSDASNEAILI
YFYCGCICCELFLYCCFGNAVTEQSKTLPIRSFNSSWYDYDGPYRKSLLVFLTNAQQPFV
FTAGRFMDLSLPSFLGILSKSYSYIALLRQLYGPLNLGAQAPGTVVFMYFLAAFPLSSIM
S
>GmmOR18 TMP_Or18-RA:CDS scf-648792 266134:274630 forward
MAANLREELERSRLSHQKILKPLKVISLAVGVDIRQPTRFWNPVKILAIALIVNGFVGFI
ADYNFIRNNLNESINVYADSLLISMQIIISNIKLIHLILKQHEFYKVIKMAENSEILLNL
REFELTTNEHKNLMQKIKDILKESWQDIHRQLSFFIYSCCAIVGWYWIVALAQNIHDLYK
RSEHFTVTTTHPVKYPVWINKGPKFIIDPLQYINSAACNHISGFGAVCYDGIYVVLVVHC
AALVHVLRVLVEHSTTNEIPQARRVQYLLGCIRFYQKIFEFYSSIDGLFRIVNLIQYCIN
AIILCMIIFQASIGLEAEASLVVKMFLYLLAIGFQNIMYCYNGEKLITQSNSLPIAWYSC
CWYNESAEFKFLVRMMILRTNRALYMKMSDFSTMSLMTMLTVIISDKLLLFFTLEKRQCG
SMKAYAVQL
>GmmOR19 GMOY012322 (TMP008029) scf-648792 271039:283655 forward
MSNMLIEVLKNEQKCNNKTLKNLYKISFMTGVNIKYKTDFKDPVKLVNLFLITISLMGLC
AQYCLVWHNRHESFVESADAICTANQAWISIFKMIYLVFVQHKFYDLLHAATDGNLLYDL
GIFDLAINCKEQLLKEIKDILRDSWLDIRRQLNFFIFSCMMACGFYMFSCLFVNYYYMHI
NPQNFTLQLRAVCFDGIFIVVVVHCSALFQILHQLLQHATDEDIPRSERVKYLLCCVQLH
DRIYNYYAKINSMYKNPSLAQCLLSMLVLCVVMFMANVGLEEDITLFFKMLCFLMAAGFQ
IVIYCYNGQKIITQSGMSPSSWYNCSWYNESPQFKYIINMMVLRANRTLYLQVSGFTTMS
HMTLLSIVQTSGSYFLLLRNLSGMD
>GmmOR20-pseu scf-652157 - 1785914: 1788436 MW: 36658.492
MVLISEMSYVIMATMNTEHFVKVIVNLSYIRVVGVETKTRRLYKFYPKRNTNQKAYQVML
YPRNYRRTTMFYTFIHESLILTQFAYKNDKLDSSILKLRSVERGPTSFICTAKFSWTHWH
VATISQWFAFAPDSFTTDYAFSRIKENLIPDGHYERHKNTLKDIIRYPQSLLRLSPFTCI
TEWHHIVSVAKFSPRQTNDSHSITMFASLCVINLKSTVQSIIYRKINYSIGNFFQPNVFQ
TSKLIKLLLFPGCCLVQMFLIWASFKQFHLSADIRYKKMLLVLIEHFQKPPVLKATSLLD
FTFFLLMQFF
>GmmOR21 GMOY011399 (TMP013635) scf-652170 3479799:3481562 reverse
MASNDYHGGKANILYSIRDSERLDNLFILMIKYLKLTGQIPLDLSSYIPTFLSPIERWFS
RLYGFLVLFSVLHVALFLAKNTFDILETGELEQITDSLVLTMIYLFASFSSAYWMFRQKR
LMELFQQINQLHRHHSLAGVTFVSYRCSYNLAHKAVKYWNLWCIIGVVFWALAPLCMGSH
TLPLPCWYPFDALLPFIYELVYITQFWAQFNMGLLFGNGSALFVAIIIIVLGQFDVLYCS
LKNLDSHALLMSGQTLKAIKDSQSLQDDNEREVNQYLYSTEYLSDLENLSQQILKKGDKS
FKRRLHEALIECVHLHQFILKACDTLEELFNPFCLIKSLQVTFQLCLLVFGVEGERSTV

RIVNFLQYLCLTLVEFFLFAYFGEMLRRHSVRVGDALWRSRWWLNAHLIKRDVFIFLINS
ERAVKITAGKLFTMDLDRLRSVVTQAFSFLTLLQNMAEKQENEVA
>GmmOr22-pseu scf-641538 58384:59452 MW: 35047.4
MTIKIMQSFLFPNMVLKTIGYEFAHKPTSKWLLCLKRVLTHFYCSSYSIKQMYDIARNGM
PNVPLFLRLTSSLMYTISGDVKLLNFVIHLKQSKAMFKRFQYIYPQSLEEPHSYQVNQHF
WSSWDYGILYFYLLSTFFIIVSPVTNSSIVISLSSAESWMVWRFMLRRSLYDGYGHGSRI
IDIYSLSTHSCRTILCGLLLWSATDYFGNCYNLLTTYYFKFFFQSKNIGNSAYNHRWYIA
SSSYKKSILIILIRAQKEADLNANSMQTISFEAFKILLYYRYWVRFIALLLYLELC
>GmmOR23 TMP_Or23-RA scf-652170 23283182:23286809 forward
MHLRTIEDVPLYQTSLRIMKFWSFLLQDNWRRYVCLIPYVIINTTQFLDIYHSEESIDAI
VRNAYIAVLFFNTILRAVLLCINRLDYEKFMDNIRLLYIDLMDSEDNVIRALVRDTTIAA
KYIAKINLLMGVCSCVGFVTYPIFATSRVLPFGMYVPGIDKHESPFYEIFFVLQVIITPM
GCCMYIPFTNIIVAFILFGILMCKVMQHKLKQLHNIEDEKAREVIIWCIKYQLGLINYVK
TINELTTYTYLVEFLAFGAMLCAMLFLLIIVGPNGDYRHLHIHDILTECYYSLFVAEAAY
DSDWFDFNVTTQKILHLLILRAQKPCAILVGNIYPMNLEMLQSLLNTTYSYFTLLKRLYG
>GmmOR24 GMOY010839 (TMP013060) scf-652157 888049:890329 reverse
MKLYKFEDFVRLANLFYTSLGIEPYALGQSTKWQIFCRYLIFSFQIINLSSMVVCEVTYV
FLAFRNDNNFLEATMIMSYIGFVLVGIFKMLSIWRQRSLLTTFVQELLRIFPQTPEQQRL
YNLDIYVRQSTRVTVCFSLLYMLLIWTYNLFAILQYVIYERWLMWRVVGKQLPYTMYILW
DWHDHWSYYPLYALECIAGFTSAAGQISCDLLLCAFATQLIMHYDYVSRSLAMYEVKMRQ
KFREPRKAMAEDMKFLTNIIAYHANVLSLSELMNEVFGVALFFNFMASSFVICFVGFQMT
MGADPDTLFKLFLFLFTSASQVYLISHYGQQLIDAQWYNADIGYKKMLVLIAARAQKPVE
LQATKFVLISRGTMTDVSVIKKYQKYFD
>GmmOR25 GMOY012357 (TMP008534) scf-649009 35111:37791 forward
MSADQSTSSTNFNSFVKGPKNMFKLLGIKLQKQGNLISTMYTSFVVISATIVAILQLCHI
LTSDTSDPHRVQNVVYITYFTVGLGKILNILYRRSILIECLSELEGIYPDKEMERKYSLQ
HYLKRYKRAEYFFWNFAMFLISVYNIETIVRSLLQLYLKGRYGYLLLVRIYAPFPYDGKL
LVYLCYFALGSITGAWIAIIIAAADLYLLGCVLQLCLHFELLTKQLMELDTRILTESEAI
KRLNAIAKYQSELIRLSRKVNRTFSGSMIVSLTAASFIICFLLFQLLGDVQLVDIAMVLI
LLLNESKQVLLICYCGDKLLNSSQRFNESLYMHNWVDGSVRYQHHLLFMLLYARNPIMLN
LLDITDITLVTLKEVLVVKTLGKRI
>GmmOR26 TMP_Or26-RA scf-652170 7565092:7567386 forward
MLTVREVTSFPFRFYNFVGIKLFQWDDNDTLTKREKYTLLVTLIIFVMNFVCKFSCFVLR
KYEDAQELTKLISYFGFACNGVFKMLSVWIGRKTLHAVIKDLAKNFPRTASECHEYKFYE
QYAFLKRHMYLLSLIHWSIAITFMLFPIVQSIFEYLVNFNDNGKFIYRFPYIMIYPFDHH
TPAGFIFAYITQLIGGITIHSYFCGSDCLLLATVHLVNMQFVSLAVRIKKFKPQTYEKDL
KQLRKILKLHISAHQNAKLVNDVFSISIFLNYLISIAVLVMIGVQVISGSEFWEFSKFVG
FLIASASQVYYYVCLYGSLLLDYEWYFADNRYQRMVILAIARSQRPAHLTAYKFFMISMES
FANVRLNHLTRNCFVLMTTSYQFFTLLKARMEEQN
>GmmOR27 GMOY008038 (TMP010200) scf-650866 8500:9930 reverse
MDEEVIVTIKEFFDIPLKFCLTLGIRLYKWSETERTTILQVCLLSNLLLHTSVYPFFLVI
YQIKIDPNDLLGRTSSLAISLFCVNAVSKILFVARHYKDLRKIIHKLIKYFPTTSGGQKN
FNLHYEFKTMRRVSSIMLWTHLITAVLFDFTPPITFGIEYMNSGGKKEFNFILPYGIWYP
WDHEDSAIMFVFTYMTQLLGSYVAVVSFVVPDLLLISVVALANMNFRYISKLIREFHPTG
TNEDFKSLGQILHYHDDILNMIDEVNDVFSFSVLLSFFGFGGLLCLVAFNAVVGSSMLDI
FSQTLFILSILLIMYYLSAYGTEMIRLVNGNGNEIEMKFKTRQKQTIFQSTDVSVALTDH
PWYDGSIHYQRMLPFPIARAQRPAQLLGYKFFVVSMESFQSLMTTSYQLFTLIKARYDED
N
>GmmOr28-pseu scf-650866 82839: 84829 MW: 29955.6

MNGLNYSNVPLNIIEMSHLTKTQRVSTISLAFSITVIYLMVYSIPGITMNSVCILLHFWL
KCVCMGGGEVGYLAVISFVNPDLLFISIVALAYMNFKHVSKLIQELRPEGTDEDFISLST
IHARYFNLSSVYIVQSIDNVLLVCAYGTEMIRLVNGNEIEMKFKTRQKQTIFQSTDVSVA
LTDYPWYDGSIYYQRMLPFPIARAQRPAQLLGYKFFVVSMECFQSVSRYRHRLRFRYLFI
YSLFIIPVATDNVLSIIRFD
>GmmOR29 TMP_Or29-RA scf-652141 245471:248227 reverse
MVRNSGAKPMVVLDFFKVPFLILQKFGVCVYRTSSKERLSVKEAISFAISMVQIGAYSIL
VPLFFIKTTPPTTAEFSDACIPLILFFTSIVRLTSILVNPKRIRTLVDIFQKYFPQNMEE
QKNFKVERNYKELIRVTKALAICLSLGCVLFSLAPLFNFAMAYYTIGDEAKFDYRMPYPI
WYPFKVNTPGMYAIMCSTQAFAAFSCVCAYFLPNMVLITSMMLINMNFKHLAKTVRNLTP
TNTDDDMKNLSKILGHHQDTFLLVDVTNEIFNISVFISFFSSIALLCSIGMNVLGESQPY
HIIKQSLLLVTSLFDLYYTCKYADDMKTSVRNIWLNSVYRVLTTLLVHFAARGANEVRIR
EHATRNYISKPKTKYSLDMSDALAEHPWYDGSVYYQRMLLFPMARAQRPAQLMAYKFFAV
SMESVLSVNTNIAYLTSI
>GmmOR30 TMP_Or30-RA scf-652141 215509:218352 reverse
MVRNSGAQVMVVLDFFEGPFWMFEKFGVCLYRTSSKERLSLKEAISFAISMVQIGAYSIL
VPLFFIKTTPPTTAEFSDACIPLILFFTSIVRLTSILVNPKRIRTLVDIFQKYFPQNMEE
QKNFKVERNYKELIRVTKALAICLSLGCVLFSLAPLFNFAMAYYTIDDEAKFDYRMPYPI
WYPFKVNTPGMYAIMCSTQAFAAFSCVCAYFLPNMVLITSMLLINMNFNLVDVTNEIFNI
SVFISFFSSIALLCSIGMNVLGESQPYHIIKQSLLLVSSLCDLYYTCKYADDMKTSVRNI
WLNSVYRVLTTLLVHFAARGANEVRIREHATRNYISKPKTKYVLTNSYQLLTLVKARFDV
E
>GmmOR31 TMP_Or31-RA scf-650238 26161:28277 reverse
MIPQFLRDDYPLEKHLFLIPKFALRLIGFYPESKLNTPLLCWAFFNFLLLGYGSYAEFTY
GIHYLTIDMQTALDALCPVLSSIMSFIKIFFIWWHRSEYKHLIEEVRRLTAAQSSRKNVH
IKRKFFTIATRLTALVLFFGFNTSTAYTVRLVISNTILYLNQQPMPIYPLTCILSHWHGY
ITVAGFVGADGFFLGFCFYFATLFKMLQQDLSDALAVNNCKSVNATMYLALLDKNSQAIR
CEADMVSNLTDIIRRHNEIAQLMKKFSSIMVGIVLSHFITSSLIIGTSFSGYAILVYIVH
TCAVIAEISLYCLGGTAVMECVIPSSTKSSPGIMPFQNTETTNLTHTNFQNSSSSKRLAN
EELALQAYCSQWYDYSVRIQKMILLIMVRSQQTITVKVPFLTPALPMLAAVCIVIVYPAI
LRFAGSVITLFKTTI
>GmmOR32 GMOY005084 (TMP007180) scf-648410 219323:223133 reverse
MFNPVPANDKRFHWPRQCVWLKLNGSWPLKSTKEFQSEFYTTENYSSFLYILWSWYVILS
VGVTIVYQTAFLITNFGDIMMTTENCCTTFMGALNFVRLLHMRLNQNQFRRVIEQFVNNI
WINRREHHPQVAHECENRMSTFRIMTILLSCLIAMYCLLPLIILFVDVGWNAPEKPFPYK
MLFPYDAHNGWRYAFTYVFTSYAGICVVTTLFAEDSIFGFFITYTCGKFRILHQRIDNII
SDSIEVTSTRQNDNNVQRIFEKKLNEIAYDHNKLIERFSNRLEHFFNPILLVNFLISSVL
ICMVGFQLVTGQEMFIGDYVKFIVYISSSLSQLYILCWNGDDLIQHVSRSTETAKHLYGC
NWEGTTLNIRNAKKFPKWHRAEEFHLTSIHPTNKEFRQNLQIMILCSQRPVKITALKFSI
LSLQSFTAVILSTSMSYFTLLQTVYNADQEEDHNIN
>GmmOR33 GMOY005479 (TMP007587) scf-648614 40250:49045 forward
MFEEIPLIYMNVKILKFWSFLFEKNWRRYLCLPATTFLVFTQFVYMFKTKEGIDSIIRNS
YMLVLWFNTILRAYIMIYDNDKYQQVIHELVELFYDLKKSKDDYVKDLLLEVNSKGIIMA
RGNLFLGLLTCIGFGVYPSLAVDRVLPFGSIIPGINEYSSPFYELWYIFEMCITPIGCCM
YIPYTSLIVSFILFGVVMSKTLQHRLRTLHRIANQPKLIHAEIISCIKYQKRIINFIEAV
NGLCTFIFLLEFLAFGALLCALLFLLIIVNSSGQAIIVCAYIIMILAQISALYWYANELR
QENFAIAAAAYETDWFTYDTIVQKDILLVILRAQKPCSVFIMFFKSYNEILFL
>GmmOR34 GMOY011902 (TMP014141) scf-652170 25006662:25007967 forward
MTIKIVQSLNQLKQFDDYIWFPNAVLKTIGYEFAHKPTSKWLLCLKRALTLLEISAHFYC

SSYSIKQMYDIARNGMPNVPLFLRLTSSLMYTISGDVKLLNFVIHLKQSKTMFKRFQDIY
PQYFEEPHSYRVNQHFWPSWVNVILYFYLLSTFFIILLTVGYAEAHFFYLKCDELPYSFD
HPMKLLLSLLFIAFYVWRFMLPRSLYDGYGHGSRIIDIYSLSTHSCRTILCGLLLWSATD
YFGNCYNLLTTYYVKFFFQSKNIGNSAYNHRWYIASSSYKKSILIILIRAQKEADLNANS
MQAISFEAFKIVLGAVYRAFAVFRTMLK
>GmmOR35 TMP_Or35-RA scf-648722 141939:145983 reverse
MSSHSLKLEDNPMLEINVKVWKYLSVIFPDREHAWRVYVFVLPVCVMNIMQFVYLLRMWG
DLAPFILNTFFAAAIFDALLRTCLVIINRDKFEAFLLELAEMYRDIEESKDDTYGRELLR
AATATVRKISIFNLTASFFDIIGALIYPLLCEGRVHPFGVAIPGVDMTASPIYEIFYILQ
FPTPIILTTMYMPFVSLFASFAIFAKTALKVLQHRMNSIFLYNYRTEEHQFAALTACITY
YSRIARVPFIWGDYMFLAILHEYCKCSCCYCCLINFLILTKLYFKIDSSTQIVSIVMYIL
TMLYVLFTYYWHANEVIMEVGHRLMFSLNRKRTTFYCCRVLKCLRQLMGYRGIIVVNAFI
MVGNVYPMTLATFQSLLNTSYTYFTMLRGLYG
>GmmOR36 TMP_Or36-RA scf-648722 191876:198790 reverse
MYYRLPLFSVNVKGWLYFGYIGKRNQGIKSLLIVNALLTLAAEILLYIKTEDVSNVIRDI
FKTAILFNSLVRILYVMRREEDFIQFMKGIESWYQEFKDDNDHMALAILNRLPKYTKLVT
AFGLSFGSIGAVASTITALLWHTHIFPIYVPGFDAFQSPLYEIINLWQSITMVSFVMSAY
ILFTNLFISWLIFGIGLLEILCKKFEQMSSANDAERLRNLKYLIRYHKRIIRYGEELEDL
VALISMVELILFTVMLCVLLVFFLITENFVDQIATVIYIFSIFYVLFISYWHTNAFSAES
LKIPDAAYRIDWAESGPETRKCLLILISRSQTALQISESNRYT
>GmmOR37 TMP_Or37-RA scf-648373 4213:5709 reverse
MNLLYRPKLSDGKLVRLSWPIAMFRLTHIVCWPLEDDAPRWAYVFDRFCWFLAFIVFVLT
NDAELRYLRVNMQNLDELLNGVPTYLVLIEAHIRGFTLGYRKNKFKNLLRKFYTDIYVDE
RQHPSFYKKIQPRFWPLYVFSSMYVATLTNFIVTPLALLLTRGSRELTFKMIPLFDYRYF
PIYLPCLLSNIWVGFLVVSLFSAEPNILGLVVLHLHSRYLIMNENLRKKTENLLKNPSNT
EIARRFRKIVVETIDENKRLNLFAQEIQNEFSFRIFILFSFAAMCLCAVASKVYMVSISI
GRKSLSLSNSIFFFHFNDVSQNPLGSFAYIFWMFGKIQELMIIGDLGSTIIATTDEVSTM
YYNSNWESVIARSSDSCENVRLMKLLTMAIALNRKPFYLTGLNFFTVSLNTVIKILQGAG
SYFTCLISFR
>GmmOR38 TMP_Or38-RA scf-648495 88188:91340 forward
MIGILPREWSEEHVYYSLAHTLLIAMGSMFIVTVACDLYEARQNLTLLGDDIVVIIGGSL
IMLKIYFHGQHSSEIHPIVDKMNDLHKMFAEYNGRSRLTIKRLQCSFYLFEVFAFSFYIF
LIVLFASAVMVPPLLTHHGLPYRAHFPILRWENSDQHPIGFVIAYIFQVIWTFHALLSIV
CMDSLACGIFLQTALNLKILCIRLREISAQNVEERESLNELKSLIKIHQYIINLIEKINA
CYYLNYVAQMGASTFMICLTAFEALLAQDRPMIAIKFEIYMLSAFLQLLYWCCAGNLVFF
ESLNVADAAYEIPLWYTRSKEFKMTVAFLIRRAQKPLQFRPSPLYGFNIKTFTSILSTSY
SYFALLCTMNK
>GmmOR39 GMOY004392 (TMP006475) scf-648080 333109:334447 forward
MHFYKIDEFFLILHPLQKYTRWIYFDFRRRDSSVPFEFQSMTKLKISLAFVLFELICNFI
KFAIEIRADRLSEAKQIAAVTSIALLCMIRGISLYTDRNRMLAICNDLDKIFPNTFYLQR
RMRVQKLAAFFKVRFRVLRSYLYLGLPAFASIPLIRYFLFYDRENGGRLLDEYHQHASWA
PFQLKQNNRAYPYVYVYETFLTILGFTCILTWDHIFTVTVSQLTMHFEFVNTELESLNVR
DTTRSMTSKFYWRRLKEIIVYHQHVYRLAKKLNETFNLTIFLTDIGCAGSICFHLYLIAN
SDSVLTIVTFFFPCFILIAFTFDYCQQGSRLAEASARLQTVLYNQEWYDASPTYRRLMLS
LLQYAHKPFTLNGFKLFDLDMLHFQSIMTIAYRLFAFVQTQGK
>GmmOR40 GMOY012356 TMP_Or40-RA scf-649009 40802:42730 forward
MVEIKDLQIRPEVCQNPLIVIHLKSMLLYGFIVSTEQKHKRFSLRRGAVFTISFVISCAL
IVSRGFESLAAGATSCATAFLYLSTSITIVNAFFQRARVVRMCTFLHDDINKLMELADER
EKKMFAATVKYLRYVTAILWTPSVFAGFIAYADCFYRTIFMPETVFNIPQVLRGEAEPIL

LFQLFPFGEVYDNFLVGYLGACYALFLGITIIPCWHTFITCLMKYIVIKFQIINKRLEAM
DISKLSPKFSLQPDMVDNLNEKDLNYWRLKMCEFCVQEQTKIKW
>GmmOR41 GMOY006480 (TMP008603) scf-649048 4425:5787 reverse
MPCLDRFRKFIGAASLIARFCACDMFDETYTMFNPLFMVLLGFLAIYTVCFVYTIYDGFV
TNQDWTIILQCLTIGAMALQAISKYACYYIHRTTLRKVVKYLDEIYGEYQDRGARYEYAL
KKSLQKIMKCSKITGIIYFTSLAGLIVSPFIYMLITGKRELTLIALIPGIDPKETVGYFI
HTGFHSAIISFAGVGFFASEIVMFSIFLHVLLHRDILMEKFHDLNEATAKPDVPAARTRD
LLNDIIAWHQKYNLFVKYIEELFSGVIFVHVTTSCASICSTLFCIVLKVWPFAPVLLLLN
TSMYAYCGFGQLLATTNDDVTRMIYEVCIWYRLEVKEQKMILLMLRKSQYAVELTVGKIM
PLNFNTALQLSKGIYTYLMVLINFLE
>GmmOR42 GMOY006479 (TMP008602) scf-649048 15:1464 reverse
MPPLDRFRKLIKHLRVVVRFCACDMFDETYTMFNPFFMVLLLCLAIYTVCFINTIYDGFV
INNDWTIILQCLAVGAMAVQAISKYLCYYSHRITLRKMVKHLDNIYEEYQSRGTRYEYAL
KKSLQKIVKCLKVAGGIYFTTVIGLIVLPIIYMLITGKRELSLTALIPGIDPKETVGYFI
HTGFHSAIILFSGVGFFASDTIMFSMLLHVLLHRDIMVAKFYDLNEITEQDDKSAERSRP
SLNDIIAWHQKYNLFMEYIEELFSGIIFVHITTSCVSICSTLFCIVLKVWPFAPLLLISN
TSMYAYCGFGQLLATTNDDVTRMIYEVCIWYRLEVKEQKMILLMLRKSQYAVELTVGKIM
PLNFNTALQLSKGIYTYLMVLINFLE
>GmmOR43 TMP_Or43-RA scf-651490 579:1910 forward
MSPLDHFRKLLKHMRVMVRFCGCDMFDETYNILNPVFMVVLLCLAIYTACFINTIYDGFV
INNDWTIVLQCLAVGSLAVQVIIIAKIYIYIYIYIYTYNIYMIKMVKHLDTIYDEYQSRG
TQYEYALKKSLQKIVKCSKITGIIYFTSLAGLIVSPIIYMLITGKRELTLTALIPGIDPK
ETVGYFIHTGFHCAIILFSGVGFFASDTIMFSMLLHVLLHRDIMVAKFHDLNEITEQEDK
PAERSRPLLNDIIAWHQKYNLRTYAGGYENGRSQVFPCRFVEYIEELFSGVIFVHVTTSC
ASICSTLFCIVLKVWPFAPVLLLLNTSMYGYCGFGQLLVTANEDVTRMIYEVCIWYRLKV
KEQKMILLMLRKSQNAAKLTVGKIMPLNF
>GmmOR44 GMOY006265 (TMP008385) scf-648928 37939:39536 reverse
MEHELTCIQRFQKIIKVLRICAKMCGCDILNPDYKMNFITWLLIVGVNGFFLCTIYTIYK
GMTIDHDWTVIPVCMCIIGSGIQGLAKILLVLKYRKVIVEQQYFLENVYTVYQQKTERYR
HVLNRWLEYTVKTYRICASMYVFVGTIIIGFPYIYWYIYGIRTMIMQFEIPFVDPNTHTG
YIIHTLYHFPMIAWGCLGHFMTDIYMFMFIINVPLLKDLLEQKFLDLNEILEETNESEKV
LPLLKDIFQWHLKYNEFIAGVDKIYRTIIFIEITTCGLSICCTIFSIVLDVWPAAYGYII
YLIFCLYSFCIMGTLIEISNDNVIEIIYTISHWYKLKLPEQKLILIMLIKSQKPIELTVG
QILPLSVSTALQVTKVVYSFLMLLLNLLDK
>GmmOR45 GMOY007896 (TMP010054) scf-650705 207676:209075 forward
MKLYSERYQEILNVSVTLLKLCGINIFAKNFRMNLITWFIIGIIGLYFILFTYTVYVGIQ
NDWSIVLKVITMSTTATQGLIKLQNFLCNPKTLRKLAEELEEVYLEYERRTNQKYPKYLQ
RSIALIKRINYAVLAFCLIALSTVIVWPLIFSYVKGEKLLVGSMRIPCVDDKNGWGFIIH
FALQSVMLIIGAYGNFAADSYCFLLLAHTSLFKDLLYCKFQDLNEILQQYPRNSLRSKPL
LQDILKWHQKHVLFIETASSIYFWVILVQISTAVTGIVTTLVCQFLGIYPTAVGYLIYCA
VLFYIYCGIGNLYERVNEEVINIICDSLWYELTIDEQKLILFMLHKSQTVEGLTIGNLIP
LSLNTALKLTRFMYTLSMMLLQFLD
>GmmOR46 GMOY003305 (TMP005362) scf-645804 155235:156578 reverse
MIVACVDHFRKLVRIFRFCAGICGADFGDRNYNPWNPLVFVTLSSIVFFTVCTVYTIYAG
LVYDNDWTVILQCLCLASSAFQGYPDKNRECSDDRYEARRFPLHYNSYIACAISAPLSIN
FGKRLFIMRFFIPGLDPNSTMGYFLHMLIQFMCIFFGAFGNFSGDMFFIVLVLHVPLLKD
IITEKLNELNRTQLKKRNKLLKDTFEWHQRCNLFIEDISELYSEIIFIEIFLCCLGICCT
TFSIVLSLSAWPAAPAYLLFTLVAMYVYCVLGYTIEEASDTLLRNIYTECLWYELAVQQQ
QMILLMLIKAQSPVVLNIGKVMPLSLSSALQLTKAIYSFLMMLLNFLE

>GmmGR1 GMOY007472 (TMP009619) scf-650411 214655:216066 MW: 48775.35
MAFWAAATGRTSPSKVVPVLNPNQKQFLQDEIRYREKMNILARTDAGNLTDYYVRKQETI
DDPELLDKHDSFYHTTKSLLVLFQIMGVMPIHRSPPKTNLPRTGYSWTSKQVMWACVVFA
IQTTLVVLVLRERVNKFVTNSDKRFDEAIYNVIFISLLFTNFLLPVASWRHGPQVAIFKN
MWTNYQLKFLKVTGTPIVFPNLYPLTWGLCIFSWLLSIVINLSQYFLQPDFELWYTLAYY
PIIAMLNCFCSLWYINCNAFGTASRALSDSLQTTLKGDKPAQKLTEYRHLWVDLSHMMQQ
LGRAYSNMYGMYCLVVFFTTIVATYGSFSEIIDHGATYKEVGLFVIVLYCMTLLYIICNE
AHYASQSVGLDFQTKLLNVNLTAVDSATQKEVEMFLVAIAKNPPIMNLDGYANINRELIT
SVGIT
>GmmGR2 GMOY011510 (TMP013746) scf-652170 18996209:18997720 MW: 49157.684
MDIITEKEFDSTLDSSVLTDDMEKLLKSEKRKKFIKNSNHNPDLMERLTMRAKRAKLKSR
HGQTAELYDQFYRDHKLLLKLFVILGVMPLARQAGRVTFSWKSYQMYYALVFYILTTLVV
LKVGSERIKLLHTTNEYDEYIYAIIFILFLLPHFWIPYVGWGVANQVAHYKSMWGTFQVR
YYRVTGTTLVFPTLKIAIIILFIGCVLLSILFLFSLSQLLDDFLLWHTTAYFHIITMINM
NAALFYINSKGTKIASKSLSDCFKKDMRIECNYIMIKQYRLLWLNLSEILQALGNAYART
YCTYCLFMSINITIGSYGALSEILEFGFSYKVLGLVVLTIYCASLLFIICDCAQSATSVM
AEGVQSTLLSLNLLQLDVQTQKEIDHFISAIELNPPVVNLRGYALINRELLTAVNFYLHY
IHAFI
>GmmGR3 TMP-Gr5 scf-652170 18992721:18996270 MW: 59057.336
MPSSINIYLPLDNIFKMYSVLEEKVVAENINFDLLRSDLKVNLKVDNINKHKINSMDLIV
QEKEYERFERSRLNSSDGDAVEIHDQFYRDHKLLLILFRVLAVMPILRSEPGRITFSWRS
IATVYAIIFWFFMTVVVIIVGKERIHILQTTTQFDEYIYAIIFVIYLVPHFWIPFVGWGV
ATEVAKYKTSWGAFQLRYFRVTGTSLQFPRLKSLIVVISVGCLLCAILFLLCLSFLLEGY
PLWHTLAYYHIITMINMNCALWYINSRAIKTASYSMALCFRKEVMKDCSASIISKYRFLW
LNLSELLQSLGNAYARTYSTLCIFIFVNIVIAVYGAFAEIVDHKHGYTISYKEAGLIVDV
VYCCTLLFIFCDCSHNATLGVAKGIQKTLLSINLLKADLEAKNEIDLFIVAIEMNPAIVS
LKGYVTVNRELLSGSISMITVYLLVLIQFKFSLDKSTTNDKRNGFVLTFLETNDQLMLSF
VSLGNNISKLMKNTYGYYNGKRIRLDSGFVCVNR
>GmmGR4 GMOY008001 (TMP010160) scf-650833 228463:237409 MW: 55967.164
MLNNYNRKKKHDTVFLNVKPAANGIGVRKYSNGILDRIDSGFGINYDNEKRASRHSVGTI
DSMNQQFVPNIFFRNLAPVKWFLSVIGVLPMTRPGPGSAKFTIGSLAFVYSILTFTLLTL
YVVVYVANNRIRIVTSLSGPFEEAVIAYLFLVNILPIFLIPLLWWETRKICVLLNDWDDFE
ILYYQISGHSVPLHLRRTAVTVTVALPLLSVISVIITHVTMADFQLSQVIPYCILDNLTA
MLGAWWYLICEALSRTANILAERFQKALRHIGPAAMVADYRALWLRLSKLTRDTGTATCY
TFTFLNVYLFFIITLSIYGLMSQLSEGFGIKDIGLAITAVWNVCLLFFICDQAHYASFNV
RTNFQKKLLMVELNWMNSDAQTEINMFLRATEMNPSNINCGGFFNVNRNLFKGLLTTMVT
YLVVLLQFQISIPTEIHNTNVNMTLDDDDYDDDLLTNAIVTTTTTIASIKGRKDPGGGHG
IATVAISRSLFMVRKL
>GmmGR5 GMOY004207 (TMP006286) scf-647997 53981:55829 MW: 53912.25
MSQQHQQSCSYVLLHFSLIFYLCKFLGIYPQNLSAYRNKHSLEASKNGSFLVVIVMIWII
VCYNTFILSQSEGKDHREANHNALNFVIGIFLTQIKIIVMATDQLSSICNQGRLAEFYNR
VYSIDNRLIKEGCLLNNGSLALHCYVMVVLTFLFEIIIILLTFVNLINYTQWVLVMWIFS
CLPTLYDSLDKIWFTTSLHALRIRLSALNKTLTNLKGVKEKSIFKRFYEEECNELSLLKI
DYVNGLEYLKRELNAGRVVKFKNRIRSFDLNIFEQKSFAKDFINVEKVKERLTNICQLHD
EICELVQTLHKMWTCPILALMAYGFLIFTAQLYFMYCVTQKQPIPILFRSAESLLISILF
LSYTGVKCIYPIFLSWKTVLEAKCTGIALHEYAINLNDKAVYEIVSVNHLSLKLLNHAFD
FANCGFLSLSMRTLCGLSGVITSYTIILLQFNFAAQQSKEADLMGME
>GmmGR6 GMOY011615 (TMP013853) scf-652170 14942509:14944789 MW: 50882.254

MTFWRDFIKPSDSYAAEQTLLWTTFLMGLTPIRISSSERNGERNIYISRAGYIISIVQVS
FFLYSFIYSFLMDESIVGYFFKTEISQVGDVLQKFIGMSGMLILFGVSLYKTNDLMELYH
VVAKIDNQFLGVGVEFNYRFIMKFRHSKLMFIISICSIYIGGSFWMLFHNNVWPTFQAIV
AFFVPHIFLLSVVTLNVAFIMRFTQHYDLINKVLKNLRHQWETRSIKTISHKQKSLQCLD
SFSMYTMVSKNPCDVVQESMEIHQLICEAASTANKYFTYQLLTIISIAFLIIVFDAYYVL
ETLLGKSKRETKFKTIEFVTFFSCQMMLYLVAIISIVEGSNRAIKKSEKTASIVHALLNK
AKTPDVKEKLQQFSMQLLHLKINFTAAGLFNIDRSLYFTISGALTTYLIILLQFTTNSSP
PSSSDCTFNSPVTSPIPMNSTST
>GmmGR7 GMOY006209 (TMP008329) scf-648889 6876:8857 MW: 46324.344
MDVENQNNLPLARPAYRNRWRSLFSAKGLYESLQPLFLLLYWHGLMVYSIKANADGKKEL
RHSLWSHLNVFFHLTLYIVCYIMTVMNNFESVAGYFFQSKISRFGDFMQILSGFIGVTVI
YITAIIPKHYVQNCLQFIQEIDQCLLRVGVRIMYSKILRYSCIFIVAMILIDILYTAASF
QILKSANEEPSLYLHITFILQHTVVLTAIAMFSCFTKLIEARFTMLHKKSLQCFDSFSTQ
TVASKSPCEIVQESMEIHQLICEASSMANKYFSCQLLTIISTAFLIIVFDAYYVLETLLG
KSRRESKFKTIEFVTFFSCQMILYVIAIISVVEGSNRAIKKSEKTGSIVHALLNRAKTLD
VKEKLQQFSMQLLHVKINFTAAGLFNIDRSLYFTVMICETIH
>GmmGR8 TMP-Gr4 scf-651593 207733:209113 MW: 47166.812
MYIKVRSLKSQAVRKWSGKVKHQSIRQENDVILNDLRIIMFPLKIISLIPLYGSASSYEL
GPPTKILYTIVMRFLILVIPVYNLYNLYLINSTETSNEPEWIDSSLNFLNYIITISFCTR
HYQLLEKIINGLLKFYEEMKEYGKCVDVTEARFSITFTIIMLTLQCCVVILKIVAIDVPL
AFNISIESVLYAFQNIVPDLYIIFISSLMRTIATQFSNLNNIATASTCGIYEGTIKNQIS
SNTLVLGFLLQMYRKLLRIHKFISDYCTFILLFYVGYAFFSITSKTYYIFVWIISPQEIS
QSEVSLSFIILVMHMLLLILLCRCFNLVARKVFQTTNVKYVKIPRSKFPKGYMPYQGWIR
VLVVLTTLSCTPVPNWNFKICNRTLIFIYSRTLKQFGAEHPKRTGSL
>GmmGR9 GMOY011903 (TMP014142) scf-652170 23328288:23329768 MW: 39397.14
MDIVESISVLQTVYQLANLSPWILDKKTWSFLQSRVLELYTTIVIIGSSALMLYSLFTDD
ILVKASDNEIGQTVDSVQMVGIRMAHIASVLESLIRKNDQKKFYDDVKEIDRIFESSLGI
TINNRILIIKERLNELVAALDEVNLTAEPSTPMRTRMAQNHSGHVFSPKRQQESNIENSP
LTKLVVIRNIYDRLHSQSVVINHCFGVSMLINVGNDFISITSNCYWIFINFKDFSSTVID
FLQIACSAIWSVPHLLNVLVLAFICERTVHTSVDMALILHRINSNFNNDKYGSAVTQFSL
QLLHQKLSLTAAGFFTIDSTLLYTIVGATTTYLIILIQFHLNEMKPNT
>GmmGR10 GMOY003231 (TMP005286) scf-645661 235649:237209 rev
MEFSNCDLLYDHISRLTLPSTVYHELMFASVMDKGLLILSWIYGVSPWNISRRSYCCNIL
DYGQMLMMLFFYLTCYALVNFDFFGIYQAPDCNGFCRLGNQLFLHLGCLLYLTTQLLAMK
SRKKFCKKFEQTLINMDRSLETCQLDERGEMSMKRNQSLHFLYKTAMTLGLLMLLIYEVR
QLTYFYGHWFFVPLMVTTYPYSAATVMLLQFAYYVYKISDRHQIINQFLEQINQDIVKTP
KEITPEIFNVESEIKITSSGNVQLNERHVKQTTHRREENDYNEDSMKFLESSTKRYATTT
SLTKLFKMFDSVLSLSVLTNSEYGPHCVPYMSACFVITIFCIFFQIKIYHVVGGKSRLLD
YIVFIYFIWSFVTMLVTYIVLRLCCNASGEAKQTAVIVQEIMRKRPAFMFGVDGNYNEMK
SFLLQSLHWEEYFQYNAIGLFSLDYTLIFTVRKKRSRK
>GmmGR11 TMP-Gr3 scf-652146 202807:205545 MW: 52777.453
MLQRKRNKKTFVCRATNKITDFSNESNQNRMKSLELQFHRALRPLLIISEILVCAPVGLQ
NLHNQSNYCTFCRKCLQIVWGLIAYVAVAHGSYNEYFFLSAYLPTQELPFYLSEFAFYLL
HVLLIMLASYFGRKTFTFSLTFILDFDSKLLKHFKIRMHYIDLRKFLRNHLSLNFAFFLS
AVMLGFIQRRSSLLGILTVNTSYTLPNVITQTSLIQYYALVYVVNKRMQLLHQLIEQTFQ
QSLKGNIFNVQQRLRVLRGLYADMDAYTKHLNDAFAVPLLLFFMASLKSLSFYIFTLYKW
IDQWTDLSYTVISYAIIEICWQFSRAFLILHFNQAIQNQRKQAAILFTSFSSVAERLEPT
IVLIISDQPFYCAIVYGSSELYYLWNHTVKYACSDDGKRYILGKETMCVCVLQFQNNFPF
TDVLSSLSIIHIFGAIRYHLRSFNECRSKT

>GmmGR12 TMP-Gr2 scf-650947 5268:6453 MW: 44579.047
MFEKKRKNKNFFSKATNNKILDFSKKPNQKRTNSLESKFYKALAPLLMISIILSCAPIGL
QKSIKRINHYYVYQKFLQILWGLTVYISIVLGAYHEYFLWTGVLPVVQLPFYLSEIVLYL
LHVLQIMIVSYFGRNIFIYLLESIVDFDDKLKQLKIHLHYNDLRKFLRNHLLLNFAFFLC
ATTLGYIQRSTTFLGFLSINSSYTLPNVIIQTSLIQYYAMTYVINKRTQLLYQLIDQVLK
QSPTKNVFNVQQKLRFLRRLYSDMDEYTKRLNETFSISILLYFMSSVNGISFYIFCVYKY
AIEWAETEFIWMAYAIVEICVQFARVFLLLHYNQGVQNQKKRATVLFTSFRHNNERLEPT
VSTFQFFSFANIFLN
>GmmGR13 TMP-Gr1 scf-640662 25103:26585 MW: 53170.637
MIKLVKIYFNINRAVGLFNLHYDQKKGQFISHHKPTILYCALVDTAILTLLPFITIAFLK
SNYYCESIKSIMINTSLLIITDHTTIIVIMLTTWWKRQAICRWCNAFAKMLKAYLGVSDT
YEYYDIVQRTRKQLLQKLGLSSFTIVLLYIYLYGTGKGNFCPTTNDISYVLKTLFCGLTE
LIIVLIDYNLFLGLTLMHMILQILSKKLKEISHDIQLMQRMQEIRYEMRESSKTFNPHMW
LKQLQGDVDAVAAESAKLKKMIYELVRILQVPYLCLLLNAFQSLTAIMFHILRFTQQRHL
DIITVIFYLIVLSANITNLMKSFQVCEYLCEDFKIFNEKVYALILNDNLRQAVNDNGADN
SLVLSRLFLENSSDNDSRKKDEIWFSFLTKYEKNGNLSHIPIINTILQVKLIVLETFSGK
VRNIIAIDHVKNELSTCVCMQDETMNDEFTKAKDMKI
>GmmGR14 TMP-Gr6 scf-652170 14938990:14942365 MW: 35515.863
MSTKFQRLRQHFISHQVFEALQPLFFITFLYGLTPFRVTTNKGGEKSIKMSAFGFINIAL
YILLYGSCYIISLLREESVAGYFFRNKISDVGNTMQICNGLITGTVIYISAVTQRSKMLR
VIATLHNLDKNFANIGIKVKYSRIYRFSIVMLTFKSLVIGVYFVGVYRLLMSMNITPAFT
VCVTFFLQHSVLFLAICLFCFVARSFERRLVILNKVSIGELVLCIVAELIDVLIPRLFST
MPTDTWPIREMLPSFPELITVCFSHFRRRASSEDNVLMTYIKPLQSMSFVQFFESNNLSS
FPLKINLKL
