# Supplementary material for: Odorant and Gustatory Receptors in the Tsetse Fly Glossina morsitans morsitans
Source: PLoS Negl Trop Dis. 2014 Apr 24;8(4):e2663. doi: 10.1371/journal.pntd.0002663 (PMC3998910; doi:10.1371/journal.pntd.0002663)

Figure S1: The genomic structure of Glossina morsitans morsitans Chemoreceptor OR and GR genes. The horizontal bold type line represent the scaffold sequence, with numerical sequences indices; yellow bars are the coding exons for each gene locus; gene names annotated are indicated at the 5' ends of the genes

Scf-639717: gene loci with alternative splice variants

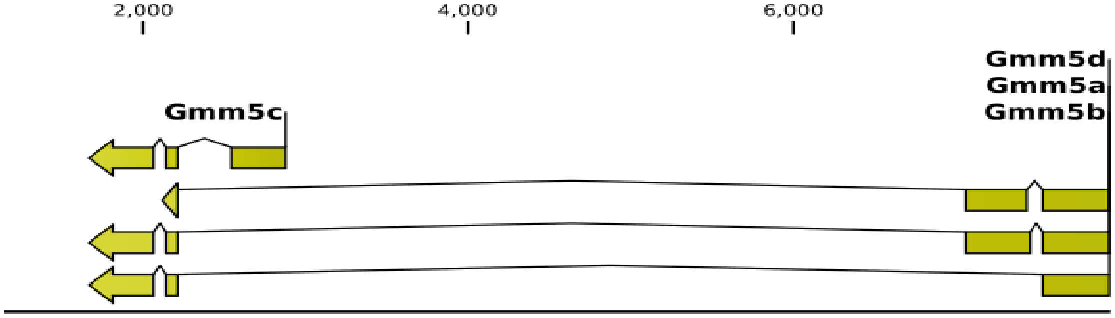

scf-648756

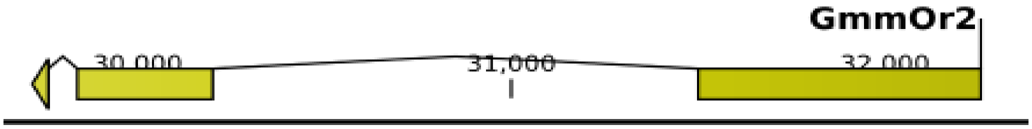

Scf-644232

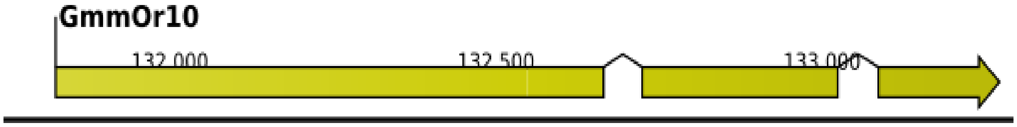

Scf-651846

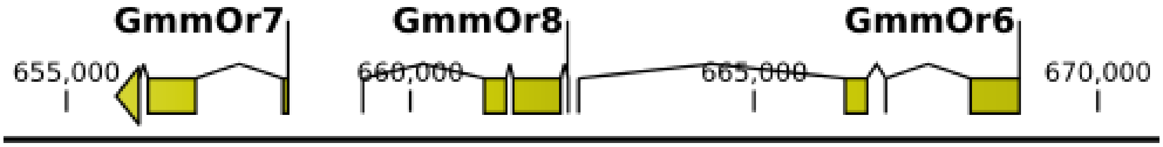

Scf-648410

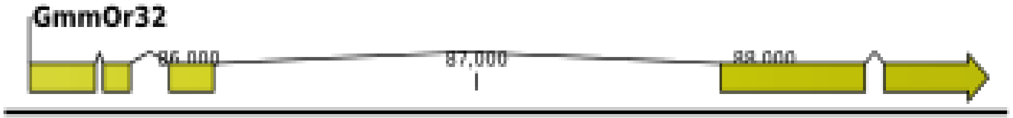

Scf-645812

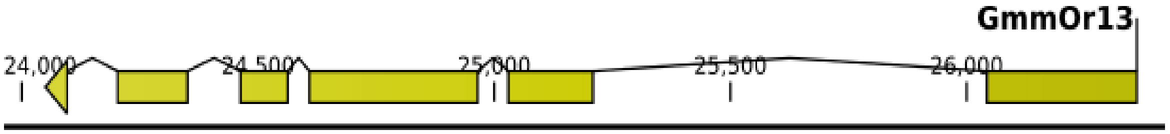

Scf-648495

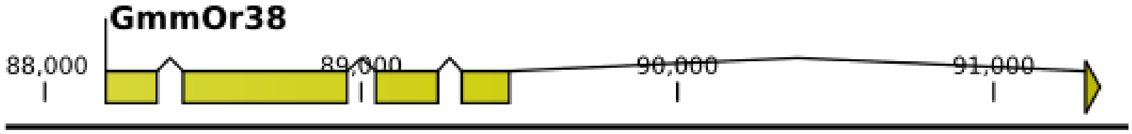

Scf-648373

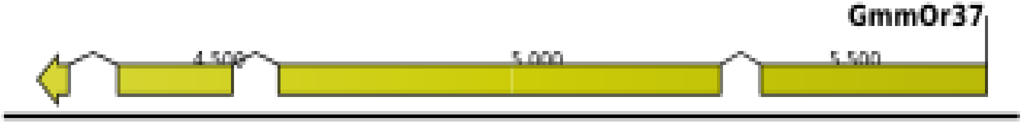

Scf-648373

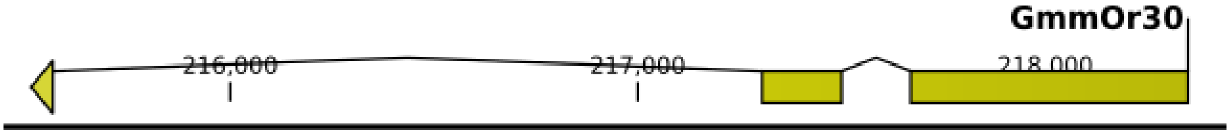

Scf-648080

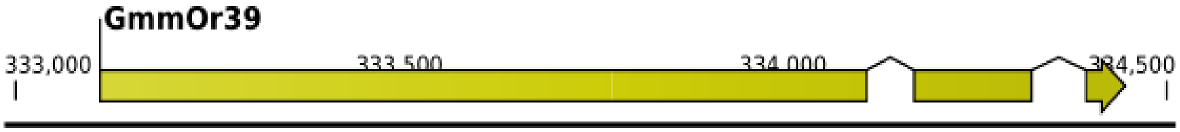

Scf-648614

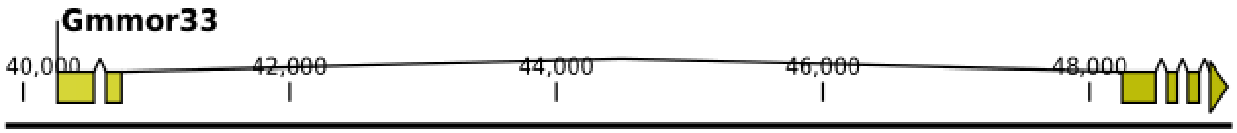

Scf-648722

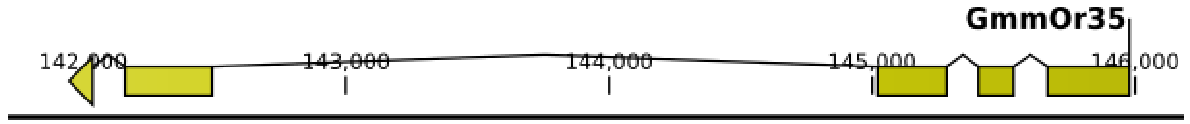

Scf-648722

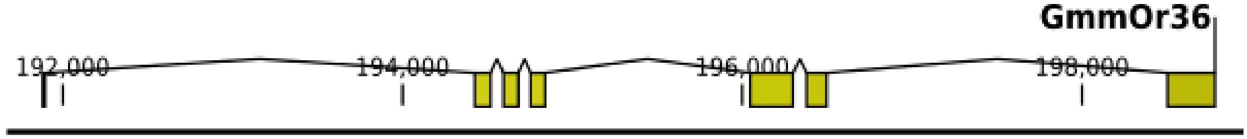

Scf-648792: gene loci in tandem

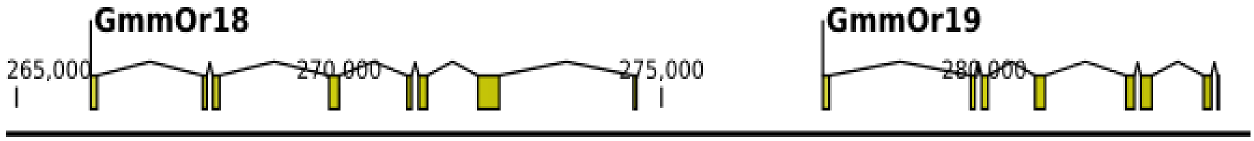

Scf-649009: gene loci in tandem

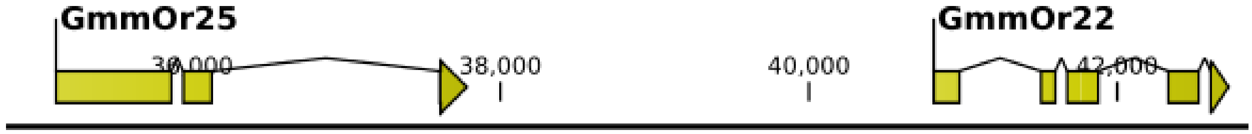

Scf-649095

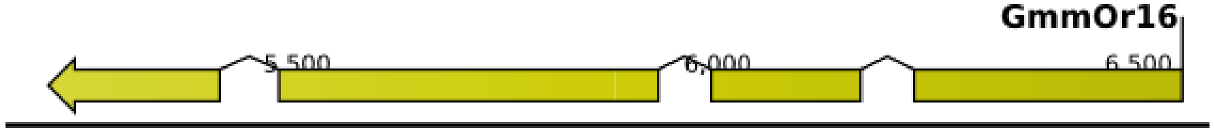

Scf-650238

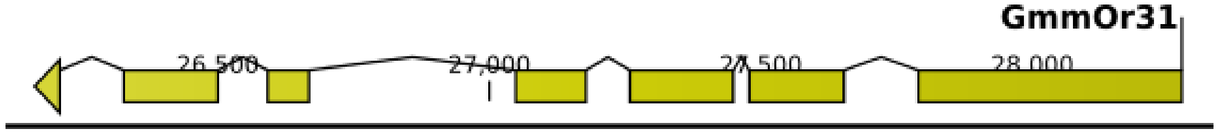

Scf-651490

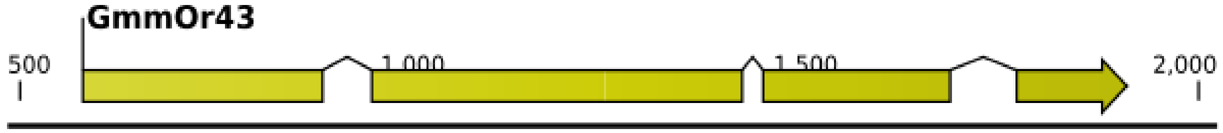

Scf-650866: gene in tandem; Or28 pseudo gene

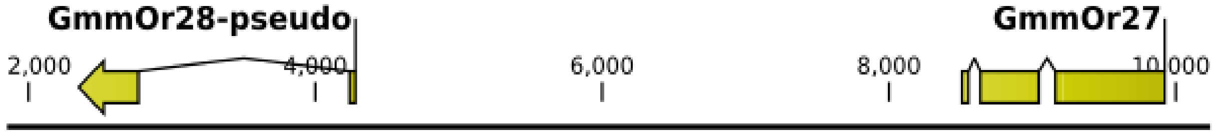

Scf-651831

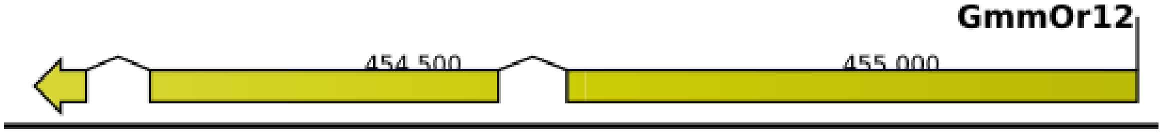

Scf-651027

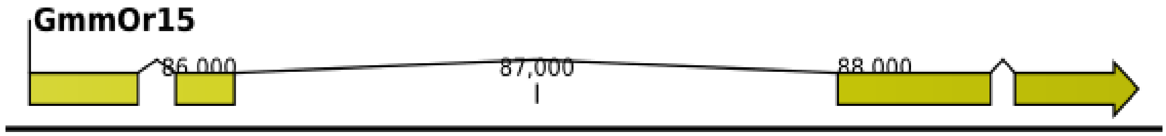

Scf-652141

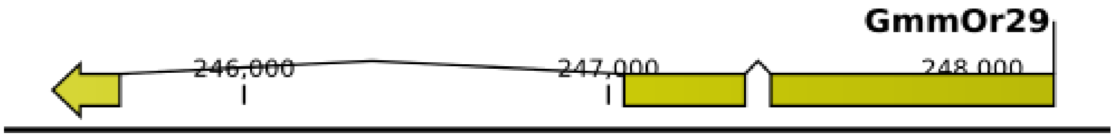

Scf-651846

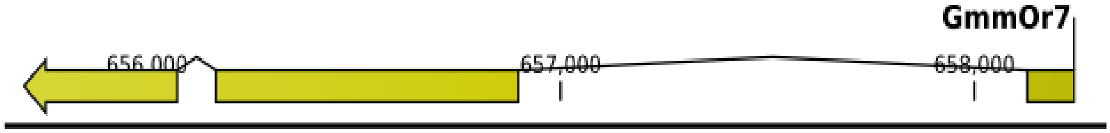

Scf-652156

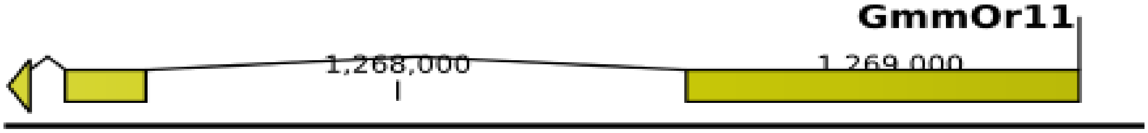

Scf-652157

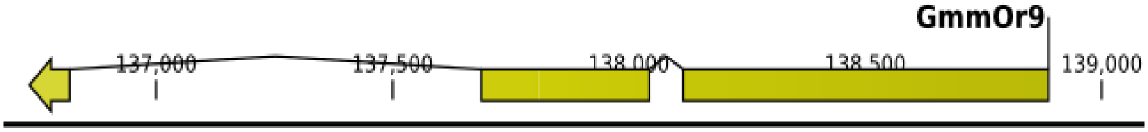

Scf-652170

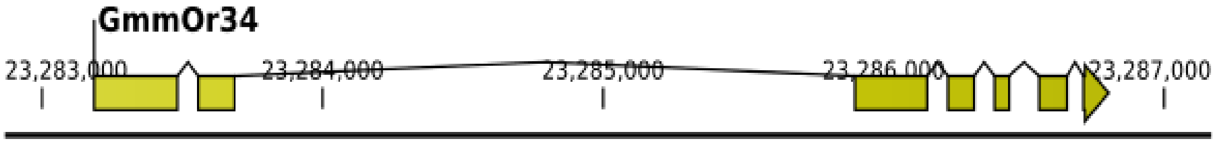

Scf-652157: GmmOr20 is a pseudo gene

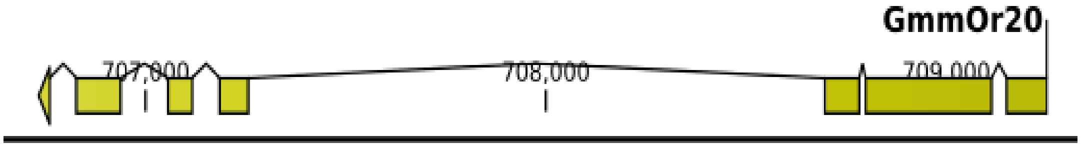

Scf-652170

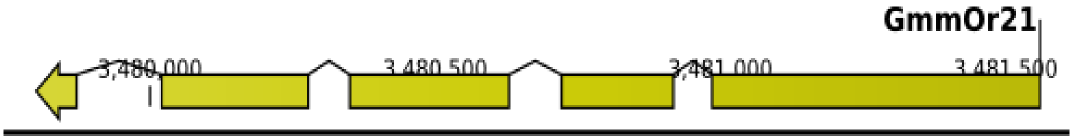

Scf-652170: GmmOr23 has only 2 THMM, likely pseudogenization

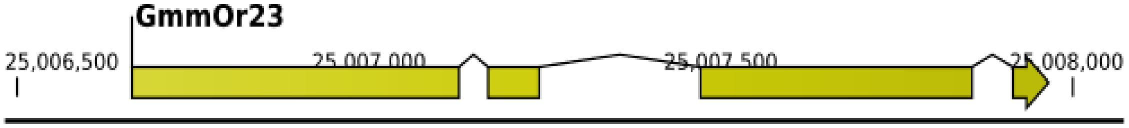

Scf-652157

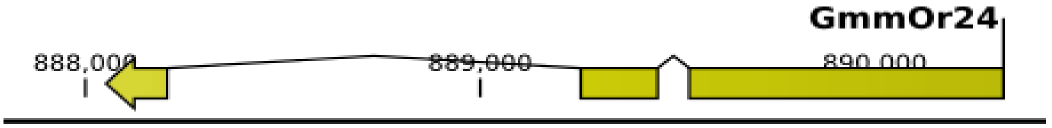

Scf-649048: genes in tandem

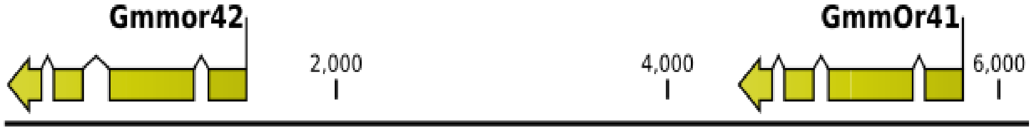

Scf-652170

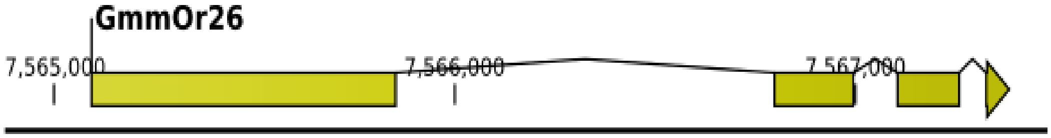

Scf-650866: pseudogene

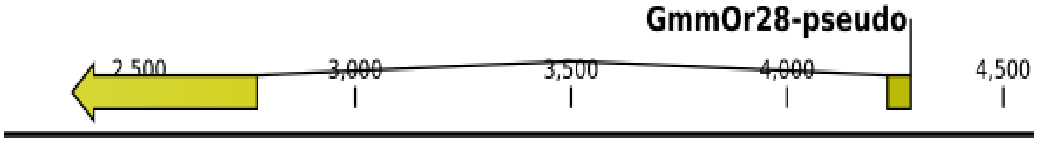

Scf-648928

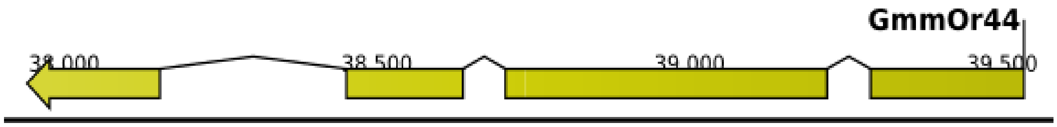

Scf-650705

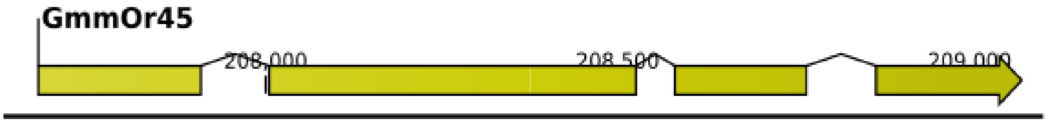

Supplement: Figure S1 — Glossina m. morsitans ORs and GRs genome structure. Most of the gene loci were encoded as singlets on their respective scaffolds. Some loci were encoded in tandem in their respective scaffolds suggestive of possible joint expression regulation. All genes had multiple exons ranging from two to eight or 12 in GRs or ORs respectively. (PDF) [file pntd.0002663.s002.pdf]
