# Supplementary material for: Odorant and Gustatory Receptors in the Tsetse Fly Glossina morsitans morsitans
Source: PLoS Negl Trop Dis. 2014 Apr 24;8(4):e2663. doi: 10.1371/journal.pntd.0002663 (PMC3998910; doi:10.1371/journal.pntd.0002663)
Supplement: Table S1 — Reciprocal blast of G. m. morsitans ORs and GRs to non-redundant protein database at NCBI. The names of annotated gene loci, scaffold identity, gene location within the scaffold, in bracket (*) refers to the coding strand where (−), reverse and (+), forward strands. The reciprocal blast gives the distribution of homology to other insects including D. melanogaster. (PDF) [file pntd.0002663.s003.pdf]

Table S1. The genomic annotation of *Glossina morsitans morsitans* OR and GR genes.

<table>
<tr><th>G. m. morsitans</th><th>Scaffold</th><th>Gene region</th><th colspan="3">Reciprocal BlastP search</th></tr>
<tr><th></th><th></th><th></th><th>NCBI acc. no.</th><th>E-value</th><th>Description</th></tr>
<tr><td>GmmOr1</td><td>scf7180000648683</td><td>14760..27560 (-)</td><td>AFH96943.1</td><td>0.00E+000</td><td>odorant receptor co-receptor [Chrysomya rufifacies]</td></tr>
<tr><td>GmmOr2</td><td>scf7180000648756</td><td>29714..32260 (-)</td><td>NP_525046.1</td><td>5.00E-126</td><td>odorant receptor 2a [Drosophila melanogaster] >sp|O46077.2|</td></tr>
<tr><td>GmmOr3</td><td>scf7180000648228</td><td>109437..110684 (-)</td><td>XP_002100282.1</td><td>1.80E-088</td><td>GE16253 [Drosophila yakuba] >gb|EDX01390.1|</td></tr>
<tr><td>GmmOr4</td><td>scf7180000642438</td><td>111181..112437 (+)</td><td>XP_002088410.1</td><td>7.20E-080</td><td>GE18551 [Drosophila yakuba] >gb|EDW88122.1|</td></tr>
<tr><td>GmmOr5a</td><td>scf7180000639717</td><td>1658..7954 (-)</td><td>XP_004530390.1</td><td>5.30E-070</td><td>PREDICTED: odorant receptor 33b-like [Ceratitis capitata]</td></tr>
<tr><td>GmmOr6</td><td>scf7180000651846</td><td>662439..668878 (-)</td><td>XP_002080251.1</td><td>5.00E-073</td><td>GD10347 [Drosophila simulans] >gb|EDX05836.1|</td></tr>
<tr><td>GmmOr7</td><td>scf7180000651846</td><td>655700..658243 (-)</td><td>XP_002080251.1</td><td>2.40E-078</td><td>GD10347 [Drosophila simulans] >gb|EDX05836.1|</td></tr>
<tr><td>GmmOr8</td><td>scf7180000651846</td><td>659294..662305 (-)</td><td>XP_001961367.1</td><td>3.50E-076</td><td>GF13833 [Drosophila ananassae] >gb|EDV38189.1|</td></tr>
<tr><td>GmmOr9</td><td>scf7180000652157</td><td>136730..138889 (-)</td><td>XP_002080251.1</td><td>1.90E-096</td><td>GD10347 [Drosophila simulans] >gb|EDX05836.1|</td></tr>
<tr><td>GmmOr10</td><td>scf7180000644232</td><td>131824..133273 (+)</td><td>XP_004526336.1</td><td>6.60E-104</td><td>PREDICTED: odorant receptor 46a, A-like [Ceratitis capitata]</td></tr>
<tr><td>GmmOr11</td><td>scf7180000652156</td><td>1267206..1269387 (-)</td><td>XP_004526336.1</td><td>2.20E-074</td><td>PREDICTED: odorant receptor 46a, A-like [Ceratitis capitata]</td></tr>
<tr><td>GmmOr12</td><td>scf7180000651831</td><td>454103..455258 (-)</td><td>XP_002098364.1</td><td>9.00E-087</td><td>GE10342 [Drosophila yakuba] >gb|EDW98076.1|</td></tr>
<tr><td>GmmOr13</td><td>scf7180000645812</td><td>24048..26362 (-)</td><td>XP_002019305.1</td><td>8.00E-117</td><td>GL12310 [Drosophila persimilis] >gb|EDW37939.1|</td></tr>
<tr><td>GmmOr14</td><td>scf7180000641298</td><td>67504..69789 (+)</td><td>XP_004534467.1</td><td>3.00E-043</td><td>PREDICTED: putative odorant receptor 45a-like [Ceratitis capitata]</td></tr>
<tr><td>GmmOr15</td><td>scf7180000651027</td><td>85495..88783 (+)</td><td>XP_001361066.3</td><td>1.30E-040</td><td>Or45a [Drosophila p. pseudoobscura] >gb|EAL25642.3|</td></tr>
<tr><td>GmmOr16</td><td>scf7180000649095</td><td>5203..6553 (-)</td><td>XP_004534466.1</td><td>2.20E-054</td><td>PREDICTED: putative odorant receptor 45a-like [Ceratitis capitata]</td></tr>
<tr><td>GmmOr17</td><td>scf7180000648564</td><td>107291..115929 (+)</td><td>XP_002135335.1</td><td>2.10E-044</td><td>odorant receptor N [Drosophila p. pseudoobscura] >gb|EDY73962.1|</td></tr>
<tr><td>GmmOr18</td><td>scf7180000648792</td><td>266134..274630 (+)</td><td>ACO83222.1</td><td>1.00E-106</td><td>putative odorant receptor [Stomoxys calcitrans]</td></tr>
<tr><td>GmmOr19</td><td>scf7180000648792</td><td>277481..283655 (+)</td><td>ACO83222.1</td><td>0.00E+000</td><td>putative odorant receptor [Stomoxys calcitrans]</td></tr>
<tr><td>GmmOr20</td><td>scf7180000652157</td><td>706731..709253 (-)</td><td>XP_004518350.1</td><td>9.90E-008</td><td>PREDICTED: putative odorant receptor 85d-like [Ceratitis capitata]</td></tr>
<tr><td>GmmOr21</td><td>scf7180000652170</td><td>3479799..3481562 (-)</td><td>XP_001994082.1</td><td>6.90E-037</td><td>GH22929 [Drosophila grimshawi] >gb|EDV94818.1|</td></tr>
<tr><td>GmmOr22</td><td>scf7180000641538</td><td>58384..59452 (+)</td><td>AAD26356.1</td><td>4.80E-012</td><td>odorant receptor DOR62 [Drosophila melanogaster]</td></tr>
<tr><td>GmmOr23</td><td>scf7180000652170</td><td>25006662..25007967 (+)</td><td>XP_002036307.1</td><td>5.00E-067</td><td>GM17404 [Drosophila sechellia] >gb|EDW52230.1|</td></tr>
<tr><td>GmmOr24</td><td>scf7180000652157</td><td>888049..890329 (-)</td><td>XP_001359521.1</td><td>3.10E-040</td><td>Or85b [Drosophila p. pseudoobscura] >gb|EAL28667.1|</td></tr>
<tr><td>GmmOr25</td><td>scf7180000649009</td><td>35111..37791 (+)</td><td>XP_002047978.1</td><td>2.50E-038</td><td>GJ11611 [Drosophila virilis] >gb|EDW70320.1|</td></tr>
<tr><td>GmmOr26</td><td>scf7180000652170</td><td>7565092..7567386 (+)</td><td>XP_004535567.1</td><td>1.00E-061</td><td>PREDICTED: putative odorant receptor 67c-like [Ceratitis capitata]</td></tr>
<tr><td>GmmOr27</td><td>scf7180000650866</td><td>8500..9930 (-)</td><td>XP_004535566.1</td><td>7.20E-063</td><td>PREDICTED: unknown protein LOC101461468 [Ceratitis capitata]</td></tr>
<tr><td>GmmOr28</td><td>scf7180000650866</td><td>2343..4333 (-)</td><td>XP_004535567.1</td><td>6.10E-014</td><td>PREDICTED: putative odorant receptor 67c-like [Ceratitis capitata]</td></tr>
<tr><td>GmmOr29</td><td>scf7180000652141</td><td>245471..248227 (-)</td><td>XP_004535567.1</td><td>5.70E-037</td><td>PREDICTED: putative odorant receptor 67c-like [Ceratitis capitata]</td></tr>
<tr><td>GmmOr30</td><td>scf7180000652141</td><td>215509..218352 (-)</td><td>XP_321153.1</td><td>1.40E-115</td><td>AGAP001912-PA [Anopheles gambiae str. PEST] >gb|EAA01023.2|</td></tr>
<tr><td>GmmOr31</td><td>scf7180000650238</td><td>26161..28277 (-)</td><td>NP_523470.3</td><td>1.00E-039</td><td>odorant receptor 24a [Drosophila melanogaster] >sp|P81913.4|</td></tr>
<tr><td>GmmOr32</td><td>scf7180000648410</td><td>219323..223133 (-)</td><td>XP_002075406.1</td><td>2.60E-073</td><td>GK17746 [Drosophila willistoni] >gb|EDW86392.1|</td></tr>
</table>

GmmOr33	scf7180000648614	40250..49045 (+)	NP_523721.1	3.00E-067	odorant receptor 49b [Drosophila melanogaster] >sp|Q9V6H2.1|
GmmOr34	scf7180000652170	23283182..23286806 (+)	CBA13932.1	5.50E-022	odorant receptor 85d [Drosophila melanogaster]
GmmOr35	scf7180000648722	141942..145983 (-)	ADK48351.1	1.30E-067	odorant receptor 43a [Drosophila melanogaster]
GmmOr36	scf7180000648722	191876..198790 (-)	XP_004534194.1	1.10E-039	PREDICTED: putative odorant receptor 30a-like [Ceratitis capitata]
GmmOr37	scf7180000648373	4213..5709 (-)	XP_004531513.1	1.90E-118	PREDICTED: putative odorant receptor 74a-like [Ceratitis capitata]
GmmOr38	scf7180000648495	88188..91340 (+)	XP_004522725.1	7.70E-097	PREDICTED: odorant receptor 47b-like [Ceratitis capitata]
GmmOr39	scf7180000648080	333109..334447 (+)	XP_004536014.1	5.10E-081	PREDICTED: putative odorant receptor 88a-like [Ceratitis capitata]
GmmOr40	scf7180000649009	40802..42730 (+)	XP_002049113.1	1.20E-075	GJ21406 [Drosophila virilis] >gb|EDW60306.1|
GmmOr41	scf7180000649048	4425..5787 (-)	XP_002094291.1	1.50E-074	GE21738 [Drosophila yakuba] >gb|EDW94003.1|
GmmOr42	scf7180000649048	15..1464 (-)	XP_002094291.1	1.30E-080	GE21738 [Drosophila yakuba] >gb|EDW94003.1|
GmmOr43	scf7180000651490	579..1910 (+)	XP_002094291.1	9.00E-058	GE21738 [Drosophila yakuba] >gb|EDW94003.1|
GmmOr44	scf7180000648928	37939..39536 (-)	XP_002069080.1	5.40E-095	GK24034 [Drosophila willistoni] >gb|EDW80066.1|
GmmOr45	scf7180000650705	207676..209075 (+)	ADG96063.1	3.00E-089	putative odorant receptor [Stomoxys calcitrans]
GmmOr46	scf7180000645804	155235..156578 (-)	XP_002047834.1	1.30E-084	GJ13657 [Drosophila virilis] >gb|EDW70176.1|
GmmGR1	scf7180000650411	214655..216066 (+)	AFH96947.1	6.00E-092	gustatory receptor 1 [Chrysomya megacephala]
GmmGR2	scf7180000652170	18996209..18997720	XP_312786.1	4.00E-039	AGAP003098-PA [Anopheles gambiae str. PEST] >gb|EAA08342.2|
GmmGR3	scf7180000652170	18992721..18996270	XP_312786.1	2.00E-059	AGAP003098-PA [Anopheles gambiae str. PEST] >gb|EAA08342.2|
GmmGR4	scf7180000650833	228463..237409 (-)	AFH96945.1	2.00E-087	gustatory receptor 2 [Chrysomya megacephala]
GmmGR5	scf7180000647997	53981..55829 (+)	XP_001354191.2	3.00E-045	Gr66a [Drosophila p. pseudoobscura] >gb|EAL31243.2|
GmmGR6	scf7180000652170	14942509..14944789	NP_995642.1	7.00E-058	gustatory receptor 28b, isoform C [Drosophila melanogaster]
GmmGR7	scf7180000648889	6876..8857 (+)	NP_995642.1	7.00E-038	gustatory receptor 28b, C-like [Drosophila melanogaster]
GmmGR8	scf7180000651593	207733..209113 (+)	XP_001968324.1	2.00E-027	GG24573 [Drosophila erecta] >gb|EDV57383.1|
GmmGR9	scf7180000652170	23328288..23329768	XP_002021945.1	5.00E-046	GL14254 [Drosophila persimilis] >gb|EDW25886.1|
GmmGR10	scf7180000645661	235649..237209 (+)	XP_001962923.1	1.00E-032	GF14188 [Drosophila ananassae] >gb|EDV32144.1|
GmmGR11	scf7180000652146	594756..597494 (-)	XP_309027.2	8.00E-024	AGAP006716-PA [Anopheles gambiae str. PEST] >gb|EAA45495.2|
GmmGR12	scf7180000650947	5268..6453 (+)	XP_002087574.1	6.00E-022	GE17747 [Drosophila yakuba] >gb|EDW87286.1|
GmmGR13	scf7180000640662	25103..26585 (+)	XP_309026.1	9.00E-024	AGAP006717-PA [Anopheles gambiae str. PEST] >gb|EAA45494.1|
GmmGR14	scf7180000652170	14938990..14942365	XP_002018685.1	3.00E-015	Gr28b [Drosophila p. pseudoobscura] >gb|EDW36881.1|

The Glossina morsitans morsitans annotated gene loci names, scaffold identity, gene location within the scaffold, in bracket (*) refers to the coding strand where (-), reverse and (+), forward strands; columns under reiterative blast searches gives best Diptera query orthologs for Drosophila, Anopheles and Aedes and their e-values
