# Supplementary material for: Odorant and Gustatory Receptors in the Tsetse Fly Glossina morsitans morsitans
Source: PLoS Negl Trop Dis. 2014 Apr 24;8(4):e2663. doi: 10.1371/journal.pntd.0002663 (PMC3998910; doi:10.1371/journal.pntd.0002663)
Supplement: Table S2 — List of Glossina m. morsitans gene names and their associated identities. The vectorbase identities (GMOY….) has since replaced the Glossina community annotation identities (TMP0….) that were computationally derived. Both annotation identities and phylomedb identities can be used as seed queries to retrieve their related Glossinia phylomedb 182 trees (www.phylomedb.org/?q=user/28). (PDF) [file pntd.0002663.s004.pdf]

Table S2: List of Glossina m. morsitans gene names and their associated identities

Names	Vectorbase IDs	Annotation IDs	PhylomeDB IDs
GmmOR1	GMOY005610	TMP007718	Phy004DJN0
GmmOR2	GMOY005796	TMP007907	Phy004DLA7
GmmOR3	GMOY004772	TMP006862	Phy004DLVK
GmmOR4	TMP_OR4	new	NI
GmmOR5	GMOY012018	TMP002414	Phy004DJ9V
GmmOR6	GMOY009475	TMP011670	Phy004DNDR
GmmOR7	TMP_OR7	new	NI
GmmOR8	TMP_OR8	new	NI
GmmOR9	TMP_OR9	new	NI
GmmOR10	TMP_OR10	new	NI
GmmOR11	GMOY010761	TMP012981	Phy004DNR0
GmmOR12	GMOY009271	TMP011461	Phy004DJ4E
GmmOR13	GMOY003312	TMP005369	Phy004DJWZ
GmmOR14	GMOY001365	TMP003388	Phy004DHFZ
GmmOR15	TMP_OR15	new	NI
GmmOR16	TMP_OR16	new	NI
GmmOR17	GMOY005386	TMP007492	Phy004DING
GmmOR18	TMP_OR18	new	NI
GmmOR19	GMOY012322	TMP008029	Phy004DFIF
GmmOR20	TMP_OR20	new	NI
GmmOR21	GMOY011399	TMP013635	Phy004DG76
GmmOR22	TMP_OR22	new	NI
GmmOR23	TMP_OR23	new	NI
GmmOR24	GMOY010839	TMP013060	Phy004DLG6
GmmOR25	GMOY012357	TMP008534	Phy004DLOY
GmmOR26	TMP_OR26	new	NI
GmmOR27	GMOY008038	TMP010200	Phy004DHF8
GmmOR28	TMP_OR28	new	NI
GmmOR29	TMP_OR29	new	NI
GmmOR30	TMP_OR30	new	NI
GmmOR31	TMP_OR31	new	NI
GmmOR32	GMOY005084	TMP007180	Phy004DLCW
GmmOR33	GMOY005479	TMP007587	Phy004DLQX
GmmOR34	GMOY011902	TMP014141	NI
GmmOR35	TMP_OR	new	NI
GmmOR36	TMP_OR36	new	NI
GmmOR37	TMP_OR37	new	NI
GmmOR38	TMP_OR38	new	NI
GmmOR39	GMOY004392	TMP006475	Phy004DLNZ
GmmOR40	GMOY012356	new	NI
GmmOR41	GMOY006480	TMP008603	Phy004DL5M
GmmOR42	GMOY006479	TMP008602	Phy004DGCY

GmmOR43	TMP_OR43	new	NI
GmmOR44	GMOY006265	TMP008385	Phy004DKY7
GmmOR45	GMOY007896	TMP010054	Phy004DKY6
GmmOR46	GMOY003305	TMP005362	Phy004DHGB
GmmGR1	GMOY007472	TMP009619	Phy004DNCP
GmmGR2	GMOY011510	TMP013746	Phy004DKZ0
GmmGR3	TMP_GR5	new	NI
GmmGR4	GMOY008001	TMP010160	Phy004DN6A
GmmGR5	GMOY004207	TMP006286	Phy004DF12
GmmGR6	GMOY011615	TMP013853	Phy004DG8D
GmmGR7	GMOY006209	TMP008329	Phy004DL2A
GmmGR8	TMP_GR4	new	NI
GmmGR9	GMOY011903	TMP014142	Phy004DMN8
GmmGR10	GMOY003231	TMP005286	Phy004DFZC
GmmGR11	TMP_GR3	new	NI
GmmGR12	TMP_GR2	new	NI
GmmGR13	TMP_GR1	new	NI
GmmGR14	TMP_GR6	new	NI

Vectorbase identities prefixed 'TMP_' are newly created during annotation and are yet to be integrated into both vectorbase and phylomedb; NI – not integrated
